# Decreased Gene Expression of Antiangiogenic Factors in Endometrial Cancer: qPCR Analysis and Machine Learning Modelling

**DOI:** 10.3390/cancers15143661

**Published:** 2023-07-18

**Authors:** Luka Roškar, Marko Kokol, Renata Pavlič, Irena Roškar, Špela Smrkolj, Tea Lanišnik Rižner

**Affiliations:** 1 Department of Gynaecology and Obstetrics, Faculty of Medicine, University of Ljubljana, 1000 Ljubljana, Sloveniaspela.smrkolj@mf.uni-lj.si (Š.S.); 2 Division of Gynaecology and Obstetrics, General Hospital Murska Sobota, 9000 Murska Sobota, Slovenia; 3 Faculty of Electrical Engineering and Computer Science, University of Maribor, 2000 Maribor, Slovenia; 4 Semantika Research, Semantika d.o.o., 2000 Maribor, Slovenia; 5 Institute of Biochemistry and Molecular Genetics, Faculty of Medicine, University of Ljubljana, 1000 Ljubljana, Slovenia; 6 Division of Gynaecology and Obstetrics, University Medical Centre, 1000 Ljubljana, Slovenia

**Keywords:** endometrial cancer, angiogenic factor, tumour-adjacent tissue, machine learning, TCGA, LEP

## Abstract

**Simple Summary:**

Endometrial cancer (EC) is a prevalent gynaecological cancer, the growth and spread of which are facilitated by angiogenesis. Our study used publicly available datasets to compare the expression of angiogenesis-related genes and proteins in EC tissue and adjacent controls. We validated these findings in a cohort of 36 EC patients and built an EC-grade prediction model using machine learning. The results showed a significant up-regulation of IL8 and LEP and down-regulation of 11 other genes in EC tissue. These genes were differentially expressed in early-stage and lower-grade EC but not in more advanced forms or in patients with deep myometrial or lymphovascular invasion. Gene co-expressions were stronger in EC tissue, especially when the lymphovascular invasion was present. More extensive angiogenesis-related gene involvement was seen in postmenopausal women. Our findings suggest that angiogenesis in EC is primarily driven by reduced antiangiogenic factor expression, with altered regulation in the tumour-adjacent tissue of EC patients with less favourable prognoses.

**Abstract:**

Endometrial cancer (EC) is an increasing health concern, with its growth driven by an angiogenic switch that occurs early in cancer development. Our study used publicly available datasets to examine the expression of angiogenesis-related genes and proteins in EC tissues, and compared them with adjacent control tissues. We identified nine genes with significant differential expression and selected six additional antiangiogenic genes from prior research for validation on EC tissue in a cohort of 36 EC patients. Using machine learning, we built a prognostic model for EC, combining our data with The Cancer Genome Atlas (TCGA). Our results revealed a significant up-regulation of IL8 and LEP and down-regulation of eleven other genes in EC tissues. These genes showed differential expression in the early stages and lower grades of EC, and in patients without deep myometrial or lymphovascular invasion. Gene co-expressions were stronger in EC tissues, particularly those with lymphovascular invasion. We also found more extensive angiogenesis-related gene involvement in postmenopausal women. In conclusion, our findings suggest that angiogenesis in EC is predominantly driven by decreased antiangiogenic factor expression, particularly in EC with less favourable prognostic features. Our machine learning model effectively stratified EC based on gene expression, distinguishing between low and high-grade cases.

## 1. Introduction

Endometrial cancer (EC) is the most frequent gynaecological cancer in developed countries. Its rates are increasing with the population ageing and with the epidemy of obesity, which is a known risk factor for EC, especially in the postmenopausal population, which presents with the most EC cases [1,2]. Nevertheless, 4% of EC patients are women aged 40 years or younger with a fertility sparing preference [3]. Despite similar early symptoms, i.e., abnormal uterine bleeding and discharge, EC does not present a homogenous malignancy. The pre-treatment diagnosis is set after the endometrial biopsy and histological verification. 

Due to its histologic heterogeneity, classification into two types of EC was proposed in 1983: type I EC, which represents a more frequent, oestrogen dependent, and prognostically favourable form; and type II which includes diagnostically less favourable, oestrogen less dependent, and prognostically less favourable cases [4]. To further reduce the diagnostic and prognostic discrepancies, recent molecular classification was introduced, stratifying EC into four risk categories: POLE ultra-mutated, microsatellite instability hyper-mutated, copy-number low, and copy-number high [5,6]. This classification of endometrial cancer has been validated and incorporated in the ESMO/ESTRO risk stratification and is currently used in clinical practice to guide EC management decisions. 

A simple hysterectomy with bilateral adnexectomy is a sufficient and final treatment in early-stage EC operative therapy. On the other hand, a suspicion of advanced disease or prognostically less favourable types of EC—with positive risk factors, such as the presence of lymphovascular invasion (LVI) or deep >50% myometrial invasion (DMI)—presents the need for the further advanced retroperitoneal procedure of additional pelvic and paraaortic lymphadenectomy, up to the level of the renal artery, and in many cases also adjuvant chemo- or radiotherapy [7]. Extended treatment is also associated with increased complications and longer recovery, which can vastly affect the patient’s quality of life. A particular EC category is represented by premenopausal EC patients wishing to retain fertility. In such cases, progestin-based therapy and the hysteroscopic resection of focal EC lesions are possible in the earliest stages of well-differentiated EC; however, hysterectomy is advised once the childbearing is completed [7,8]. 

However, besides fertility-sparing preference in younger patients and the burden of common comorbidities in older patients, the decision on the extent of treatment is based mainly on the histological findings acquired via endometrial sampling, which is only a modest predictor of surgical pathology features [9]. Additional EC stratification and an individually tailored treatment approach would reduce the possibility of both EC recurrence (due to under-treatment), and an increased rate of postoperative complications (due to over-treatment). Biomarkers may be pivotal in a more precise EC prognosis, in the clinician’s decision-making process, and in an individually tailored therapeutic approach.

Angiogenesis is one of the earliest processes promoted by cancer tissue, induced by the lack of oxygen and nutrition in a rapidly growing tumour mass through released pro-angiogenic molecules and suppressed antiangiogenic molecules, commonly named angiogenic factors (AFs). Cancer cells secrete AFs to the surrounding tissue, which provokes the growth of new vessels [10,11,12,13] and enables further cancer growth and metastasis. Our recent data and studies of other groups on preoperative plasma samples from EC patients revealed the potential of angiogenic factors (AFs) as biomarker candidates for the early diagnosis and risk stratification of EC [14,15,16]. 

Since AFs are controlled and produced directly in the cancerous tissue, confirming AFs’ expression from the tumour cell is needed. Thus, AFs gene expression levels in tumour tissue have the potential to become novel biomarkers as diagnostic and prognostic indicators of EC to guide therapies and promote an understanding of the carcinogenesis of EC. 

In recent years, artificial intelligence (AI) and machine learning (ML) methods have been ubiquitously used in several fields, including in medical diagnosis and classification tasks, and relatively recent advances in the field have allowed for the use of advanced ML methods even on (very) small datasets [17].

The aims of this study were manifold and oriented towards evaluating the diagnostic and prognostic potential of AF-encoding gene expression. (i) We analysed publicly available datasets for the expression of angiogenesis-associated genes and proteins in EC tissues (T) compared to tumour-adjacent control tissue (TA). Genes and encoded proteins with the highest change in T versus TA expression were chosen for further analysis. (ii) An additional six genes (*CSF3*, *IL8*, *LEP*, *NRP1*, *TEK*, *FST*) were included in further research based on the results from our previous studies on biomarkers in the plasma samples of EC patients [14,18]. (iii) Ultimately, 15 genes were included in the validation study using the qPCR method on a cohort of 36 EC patients. (iv) By combining TCGA data and data from our study, we applied machine learning modelling to create an EC grade prediction model based on the T gene expressions in EC.

## 2. Materials and Methods

### 2.1. Analysis of Publicly Available Datasets

We analysed publicly available datasets for the expression of 91 angiogenesis-associated genes in EC tissues compared to adjacent control tissue, and for the presence of 64 angiogenesis-associated proteins in EC tissue compared to adjacent control tissue. Details about the datasets used in this study are listed in Table 1. 

For further analysis and validation on EC tissue, genes/proteins of interest were selected when both criteria were fulfilled: more than 3-fold significant (adjusted *p* < 0.01) difference in gene expression between the tumour and normal adjacent tissue, and more than a 2-fold significant difference in protein levels between tumour and adjacent tissues. Additionally, six AFs from our previous studies [14,18]—leptin, IL-8, neuropilin-1, Tie-2, follistatin, and G-CSF—were included in the validation study in endometrial tissue. In plasma, leptin and IL-8 were significantly increased, whereas Tie-2 and G-CSF were significantly decreased in EC patients compared to control patients. Neuropilin-1 and follistatin were significantly increased in the plasma of higher-grade EC patients and LVI-positive patients, respectively. 

### 2.2. Validation Study on Endometrial Cancer Tissue

#### 2.2.1. Endometrial Tissue

The specimens of EC tissue and paired morphologically normal adjacent endometrial tissue were obtained from 36 patients undergoing hysterectomies for histologically proven EC (Table 2). All patients were treated at the Department of Gynaecology and Obstetrics at the University Medical Centre, Ljubljana, from 2003 to 2010. Samples were collected and processed according to the approved standard clinical operating procedures. All patients provided written informed consent, and the study was approved by the National Medical Ethics Committee of the Republic of Slovenia (ID 0120-429/2017/8, 5 November 2017). 

#### 2.2.2. RNA Isolation and Reverse Transcription

Immediately after surgery, the tissue samples were stored in RNAlater (Thermo Fisher Scientific, Waltham, MA, USA) at −20 °C to stabilize and protect cellular RNA. Tissues were then disrupted in the presence of liquid nitrogen using a mortar and pestle. The total RNA from tissue samples was isolated using Tri Reagent (Sigma-Aldrich, St. Louis, MO, USA), according to the manufacturer’s instructions. The RNA samples were additionally cleaned, and residual DNA was removed using RNeasy Mini kits and RNase-Free DNase sets (Qiagen, Düsseldorf, Germany), respectively. We then analysed the purity and quality of extracted RNA with the Agilent 2100 Bioanalyzer using the RNA 600 Nanokit (Agilent Technologies Inc., Santa Clara, CA, USA) and demonstrated that the RNA was of good quality (an average RIN 7.8 ± 0.80). Samples of the total RNA were reversely transcribed into cDNA using RT2 First Strand Kit (Qiagen, Hilden, Germany) according to the manufacturer’s instructions. The cDNA samples were stored at −20 °C.

#### 2.2.3. Quantitative Real-Time PCR

The expression of 15 genes encoding AFs was examined in pairs of cancer and adjacent control endometrium using TaqMan Gene Expression Assays (Applied Biosystems; Foster City, CA, USA) listed in Table 3.

The quantification was accomplished using the Applied Biosystems ViiA 7 Real-Time PCR System (Thermo Fisher Scientific, Waltham, MA, USA), as described in our previous studies [23]. Shortly, each sample was run in triplicates (replicates of 0.25 μL cDNA in a total reaction volume of 5.0 μL) using the Applied Biosystems MicroAmp Optical 384-well plates (Thermo Fisher Scientific, Waltham, MA, USA). The amplification efficiency (E) was first calculated from the slope of the log-linear portion of the calibration curve for each gene and was accounted for in further calculations. Next, the normalization factor was calculated for each sample based on the geometric mean of the two most stably expressed reference genes (*HPRT1* and *POLR2A*). Last, normalized RNA was calculated from the crossing-point value (Cq) as E^−Cq^, divided by the normalization factor. The Cq cut-off value was set to 36. We followed The Minimum Information for Publication of Quantitative Real-Time PCR Experiments guidelines in performing and interpreting qPCR reactions [24]. 

#### 2.2.4. Statistics

In the first part of the study, protein and mRNA levels were evaluated in T and TA tissue in publicly available datasets in up to 24 paired samples (all samples where the data were available for both T and TA tissue) and analysed using Wilcoxon matched pairs signed rank test with Bonferroni–Šidák corrections for multiple comparisons; an adjusted *p* level < 0.01 was considered significant. Further on, in the clinical cohort, gene expression was evaluated in 36 paired samples, which were further stratified into two groups based on the clinical data (FIGO stage, menopausal status) and the histopathological data (tumour histological grade, depth of myometrial invasion, and presence of lymphovascular invasion). mRNA expressions in the tumour samples and the matched adjacent tissues were analysed using the Wilcoxon matched-pairs signed rank test with Bonferroni–Šidák corrections for multiple comparisons. Unmatched data within tissue groups were analysed using the Mann–Whitney U test with Bonferroni–Šidák corrections for multiple comparisons. Unless noted otherwise, data are presented as mean ± sd and *p* level < 0.05 is considered significant. 

### 2.3. Machine Learning Modelling

Since the study included a very small dataset of 36 patients, as described in Section 2.2.1, the data modelling was performed by combining the data from the TCGA Pan-Cancer study (TCGA data obtained with new generation sequencing technology) and the data from our study (study data). The modelling used all available TCGA data records that contained both the tumour tissue measurements and the tumour-adjacent tissue measurements, combined with a part of the study data for training and the remaining study data for testing. The combined training dataset, therefore, included 44 records, whereas the test dataset included 14 records, which can be sufficient if adequate ML approaches are used [17].

The general steps performed were the following:

1. TCGA and study data were normalised for merging;
2. TCGA data (22 samples) were merged with 22 stratified randomly selected samples of study data; the remaining 14 samples were assigned to the test dataset;
3. An automated machine learning (AutoML) approach was used to create the models on the training dataset;
4. The models were tested on the test dataset.

#### 2.3.1. Merging and Normalisation

Combining a generally available dataset with a part of the target dataset to increase dataset size and reduce model overfitting has been described previously [25], however, with directly mergeable data. Since TCGA and study data were measured using a different approach, a normalisation process needed to be devised to allow the data to be merged. Several approaches have been studied previously, for example, combining microarray data with RNA-seq data [26] or normalising for other divergent factors in gene expression measurements [27].

However, since we wanted to preserve the information of the ratios based on the original distribution of the data (thus making, e.g., quantile normalisation less appropriate), and the data were not normally distributed (thus making e.g., Z scoring less appropriate), we devised the following normalisation method:

- The best fitting distribution for data was empirically selected by trying to fit the data to one of the common standard distributions (Normal, Log-normal, Poisson, Beta, Gamma).
- For distributions that require positive data, the data were right-shifted to ensure that the smallest value was positive.
- The best fitting distribution for most columns (Gamma) was then fitted for all columns, and distribution parameters were calculated, together with the correlation coefficient, significance, and estimated lower and upper bounds at the 95% confidence level using the MATLAB “*corrcoef*” function. It is worth noting at this point that the Gamma distribution has previously been linked to gene expression in multiple studies [28].
- The original values were then transformed to the value of the cumulative distribution function (CDF) of the fitted distribution at the original value, thus obtaining a value between 0 and 1, indicating the relative (expected) ratio of the population with a value lower than the original value [29].

Distribution fitting and transformation to the CDF values were performed using MATLAB R2022b software; the fitted parameters are available in the Tables S4 and S5. For missing data, the value of 0.5 was used, effectively meaning that median-based imputation was used for missing data imputation.

The data were then merged into a single dataset, and finally split into the training and test datasets:

- The training dataset was created by taking all 22 normalised samples from the TCGA dataset and combining them with 22 randomly selected samples from the study dataset, where a stratified random sampling approach was used to ensure the final dataset had a balanced distribution of the output variable (EC grade).
- The remaining samples from the study dataset represented the test dataset.

The training and test datasets were compared using the Wilcoxon rank-sum (Mann–Whitney U) test using MATLAB’s built-in “*ranksum*” function and were then exported into CSV files for further processing/modelling.

#### 2.3.2. Modelling and Testing

The case/control classification models were created using the previously described dataset using the mljar-supervised library 0.11.3 [30] and the underlying scikit-learn library version 1.1.1 [31], which can be used successfully for small dataset modelling, as was previously shown in similar datasets [14]. The library was configured using the “Compete” mode, and the model validation phase was customised to utilise a stratified 5-fold validation approach for model selection, where the models were optimised to improve the area under the curve (AUC) for the receiver operating characteristic (ROC) curve using the mljar-supervised, built-in roc_auc metric. The training was limited to 20 min per model.

Data were imported into Python using the Pandas library from the CSV format [32], and three subsets of the data were created based on the hypotheses tested:

- A model utilising the data combining the tumour tissue, adjacent tissue data, and calculated ratios between the tumour tissue and adjacent tissue measurements;
- A model utilising only the tumour tissue data;
- A model utilising only the adjacent tissue data.

For each feature group, the best-performing model (calculated using the aforementioned 5-fold cross-validation method within the training dataset) was tested on the study data holdout (test) samples, containing four high-grade and ten low-grade samples. Confusion matrices were generated utilising the decision threshold calculated during training, providing the four standard metrics (true positive, false positive, true negative, false negative), based on which the model precision, recall, accuracy, sensitivity, specificity, and F1 score were estimated. Confusion matrices were further tested using Fisher’s exact test to confirm that the model result was statistically significantly divergent from random guessing.

All models were trained to predict whether the sample belonged to the high-grade or low-grade EC group. The complete MATLAB, Jupyter Notebook, and Python scripts are available at the following link: https://github.com/klokedm/EndometrialCancerGradePrediction.

## 3. Results

### 3.1. Public Databases Examination Revealed Twenty-One AF-Encoding Genes and Twenty-Two AF Proteins That Fulfilled Selection Criteria; Nine Gene/Protein Pairs Were in the Intersection

**Figure 1:**
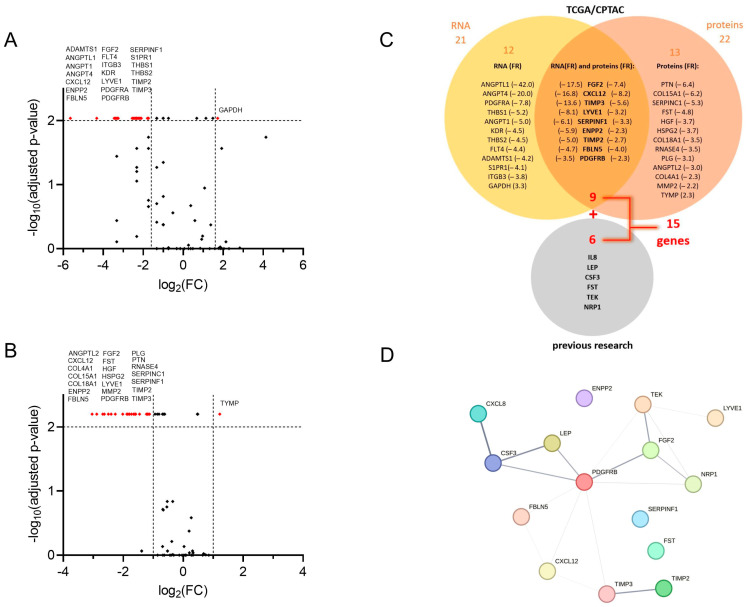
Selection of angiogenesis-related genes in the study. Volcano plot visualising fold change (FC) and the corresponding *p*-values of (**A**) normalised mRNA (data are from the GDC TCGA Endometrioid Cancer (UCEC) study, downloaded from UCSC Xena server [21]). Paired samples, *n* = up to 23; Wilcoxon matched-pairs signed rank test with Bonferroni–Šidák corrections for multiple comparisons, and (**B**) angiogenesis-associated proteins in tumour tissue versus control tissue (data are from the CPTAC UCEC Discovery Study—Proteome, PDC ID: PDC000125 [22]. Paired samples, *n* = up to 24; Wilcoxon matched-pairs signed rank test with Bonferroni–Šidák corrections for multiple comparisons. Vertical lines: log_2_FC cut-off values in a selection protocol; red dots: genes/proteins that reach more than 2- or 3-fold significant difference with adjusted *p* < 0.01 as a criterion for further evaluation. (**C**) Venn-diagram of a selection process, and 21 genes with more than 3-fold expression change in tumour versus adjacent tissue, and 22 proteins with more than 2-fold level change in tumour versus adjacent tissue. Nine proteins and their encoding genes fulfilled both criteria simultaneously and were chosen for further validation using a clinical cohort. Genes encoding six proteins from our previous research [14,18] were added, leading to further analysis of 15 genes. (**D**) Analysis of protein–protein interactions from the STRING database for association networks [33]. Several known (from curated databases) and predicted interactions (based on gene co-occurrence, co-expression, and gene homology) are shown; the line thickness indicates the strength of data support.

We first examined the publicly available mRNA and proteome datasets for the presence of angiogenesis-associated proteins (EC tissue, *n* = up to 95; TA tissue, *n* = up to 25; paired samples, *n* = up to 24) and the expression of angiogenesis-associated genes (EC tissue, *n* = up to 548; TA tissue, *n* = up to 35; paired samples, *n* = up to 23) in EC tissue compared to adjacent control tissue. The expression of 91 angiogenesis-associated genes and the levels of 64 angiogenesis-associated proteins in endometrial cancer tissues and adjacent control tissues are collected in Tables S1–S3. The most significantly down-regulated and the most significantly up-regulated genes are shown in Figure 1A, and differential levels of angiogenesis-associated proteins in EC compared to adjacent control tissue are presented in Figure 1B. 

Nine genes/proteins of interest (*CXCL12*, *ENPP2*, *FBLN5*, *FGF2*, *LYVE1*, *PDGFRB*, *SERPINF1*, *TIMP2*, *TIMP3*) were then selected from analysed datasets based on the following criteria: (a) the significant difference (adjusted *p* < 0.01) in gene expression between tumour and tumour-adjacent tissue was more than 3-fold, and (b) the significant difference in protein levels between tumour and adjacent tissues was more than 2-fold. An additional six genes (*CSF3*, *IL8*, *LEP*, *NRP1*, *TEK*, *FST*) were included in further research based on the results from our previous studies of biomarkers in plasma samples of EC patients [14,18]. We ultimately chose 15 genes, which we further examined in the validation study on a cohort of 36 EC patients (Figure 1C).

Finally, we analysed the STRING database [33] for the protein–protein interaction between proteins encoded by 15 selected genes. This way, we detected several known (i.e., from curated databases and credible experimental data) and predicted interactions (i.e., based on gene co-occurrence, co-expression, and gene homology). The interactions are depicted in Figure 1D and are ranked (the thickness of the line) based on the interaction confidence score. The highest combined score (0.913) was assigned to the interaction between CXCL8 (also known as IL-8) and CSF3, coming in the main part from the database annotation score (0.900), enhanced with the co-expression score (0.172). This is followed by LEP/CSF3 and TIMP2/TIMP3, with combined scores of 0.800 and 0.714, respectively. High scores (0.600) from the database annotations are also assigned to PDGFRB/FGF2, TEK/FGF2, PDGFRB/LEP, and PDGFRB/CSF3. TIMP2/TIMP3 and PDGFRB/TEK show the highest gene homology, with scores of 0.925 and 0.588, respectively. The gene co-expression is highest between PDGFRB and each TIMP3, CXCL12, and FBLN5 (co-expression score 0.227, 0.211, and 0.180, respectively), as well as between TEK and LYVE1 (co-expression score 0.179).

### 3.2. Validation of Findings on the Clinical Cohort

#### 3.2.1. Clinical Characteristics of Enrolled Patients

Paired samples of tumours and tumour-adjacent tissues were collected from 36 patients with histologically verified EC. Their mean age was 62.1 ± 14.0 years. In total, 25 patients (69.4%) were postmenopausal, 28 patients (77.8%) were diagnosed with endometrioid adenocarcinoma, and 8 (22.2%) with other histological types. Deep myometrial invasion was observed in nine EC patients (25.0%), <50% invasion into the myometrium in nineteen EC patients (52.8%), and no invasion into the myometrium in eight EC patients (22.2%). LVI was observed in eight patients (22.2%). According to the classification of the International Federation of Gynecology and Obstetrics [34], twenty-five patients were diagnosed with EC in stage IA (69.4%), nine patients in stage IB (25.0%), one patient in stage II (2.8%), and one patient in stage IV (2.8%). The detailed clinical characteristics are presented in Table 2.

#### 3.2.2. Thirteen Genes Encoding AFs Are Differentially Expressed in Tumour Tissue Compared to Adjacent Control Tissue in EC Patients 

**Figure 2:**
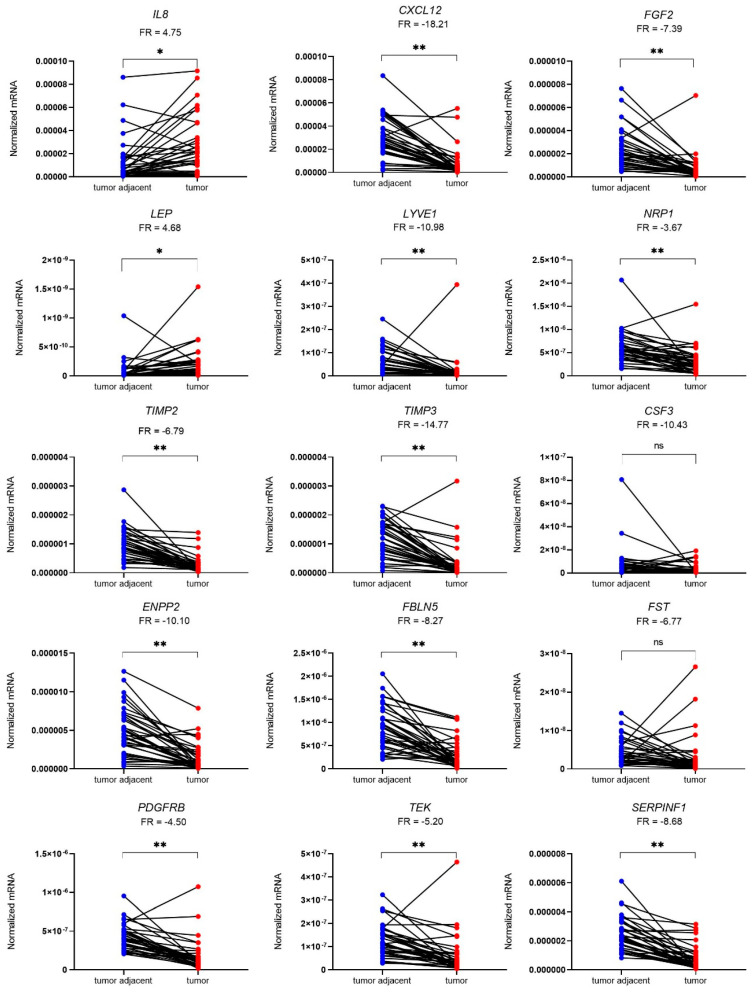
Expression of genes encoding 15 angiogenic factors in 36 paired samples of EC (T) and tumour-adjacent (TA) tissue. Data were analysed using the Wilcoxon matched-pairs signed rank test with Bonferroni–Šidák corrections for multiple comparisons. Fold regulation (FR) is presented as a mean of pairwise T/TA expression ratios or their negative inverse values. * *p*-value ≤ 0.05, ** *p*-value ≤ 0.01, ns—not significant.

The local expression of 15 genes encoding proteins involved in angiogenesis pathways was determined using quantitative polymerase chain reaction (qPCR) in 36 patient-matched samples of tumour and tumour-adjacent macroscopically normal tissue. A total of 13 genes were differentially expressed in EC versus adjacent control tissue samples. *IL8* and *LEP* were up-regulated in tumour tissue (4.8-fold and 4.7-fold, respectively), while *CXCL12*, *FGF2*, *LYVE2*, *NRP1*, *TIMP2*, *TIMP3*, *ENPP2*, *FBLN5*, *PDGFRB*, *TEK*, and *SERPINF1* were down-regulated in tumour tissue in comparison to morphologically normal tumour-adjacent tissue (Figure 2). The most profound differences in the gene expression between the two tissues were observed for *CXCL12* and *TIMP3* genes (18.2-fold and 14.8-fold change, respectively). Mean fold changes in tumour-to-adjacent tissue expression and the 95% CI for all genes are listed in Table 4. 

#### 3.2.3. Relationships of Gene Expression with Clinical Characteristics

In the next step, we analysed the effect of the clinicopathological conditions of patients on the expression of the selected genes. We stratified patients according to the clinical data, i.e., FIGO stage, menopausal status, and the histopathological data, i.e., histological tumour grade, depth of myometrial invasion, and the presence of lymphovascular invasion. 

##### In Early Stages and Lower Grades of EC, but Not in More Advanced or Aggressive Forms of EC, Genes for AFs Tend to Be Differentially Expressed in Tumour Tissue Compared to Adjacent Control Tissue

**Figure 3:**
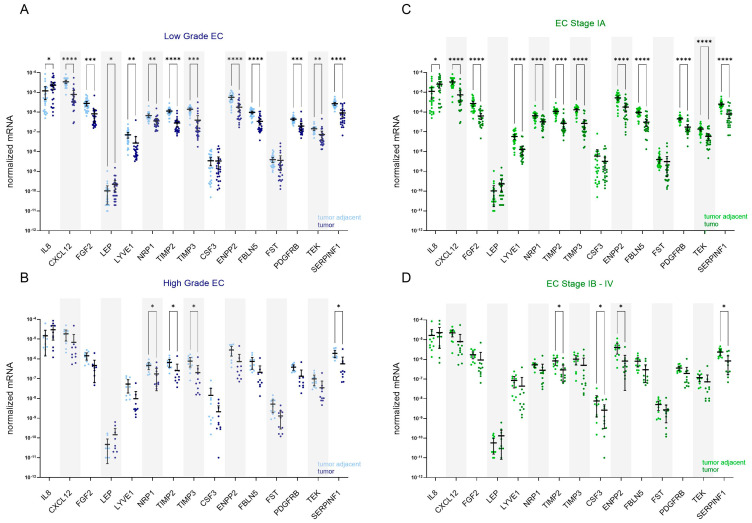
Expression of angiogenesis-related genes in EC patients stratified based on the cancer grade and stage. Expression of genes encoding 15 angiogenic factors in paired tissue samples from patients with (**A**) low-grade EC (*n* = 26) and (**B**) high-grade EC (*n* = 10); tumour tissue is shown in dark blue and tumour-adjacent tissue in light blue colour. Expression of genes in paired tissue samples from (**C**) patients with stage IA EC (*n* = 25) and (**D**) stage IB–IV EC (*n* = 11); tumour tissue is shown in dark green, and tumour-adjacent tissue is in light green colour. Wilcoxon matched pairs signed rank tests with Bonferroni–Šidák corrections for multiple comparisons. Data are shown as scattered dot plots with marked means with 95% CI, * *p*-value ≤ 0.05, ** *p*-value ≤ 0.01, *** *p*-value ≤ 0.001, **** *p*-value ≤ 0.0001.

We assessed endometrioid EC grade 3 cancers together with non-endometrioid tumours (serous and dedifferentiated EC) as high-grade EC since several important reports have firmly demonstrated that high-grade endometrioid cancers have molecular characteristics, risk factors, clinical behaviours, and prognoses overlapping with those of non-endometrioid cancers [35,36]. The low-grade EC group comprised endometrioid EC grade 1 and 2 and mucinous EC. In patients with low-grade EC (*n* = 26; Figure 3A), there was no difference in the expression of CSF3 and FST between the tumour and adjacent tissue. In addition to those two genes, in patients with FIGO stage IA (*n* = 25; Figure 3C), there was additionally no difference in the expression of LEP. In both groups of patients, IL8 was up-regulated in tumour tissue, by 5.4-fold and 5.6-fold, respectively. All other genes were down-regulated in low-grade and low-stage EC, most prominently CXCL12, which was down-regulated by 20.9-fold and 21.9-fold, respectively. In high-grade EC (*n* = 10; Figure 3B), NRP1, TIMP2, TIMP3, and SERPINF1 were significantly down-regulated in T tissue compared to TA tissue, by 6.0-fold, 6.2-fold, 17.2-fold, and 12.2-fold, respectively. In a group of patients with stages IB–IV EC (*n* = 11; Figure 3D), TIMP2, CSF3, ENPP2, and SERPINF1 were significantly down-regulated in tumour tissue, by 4.9-fold, 9.6-fold, 10.2-fold, and 9.2-fold, respectively. 

##### Genes for AFs Are Differentially Expressed between Tumour and Adjacent Control Tissue Only in Patients without DMI or LVI 

**Figure 4:**
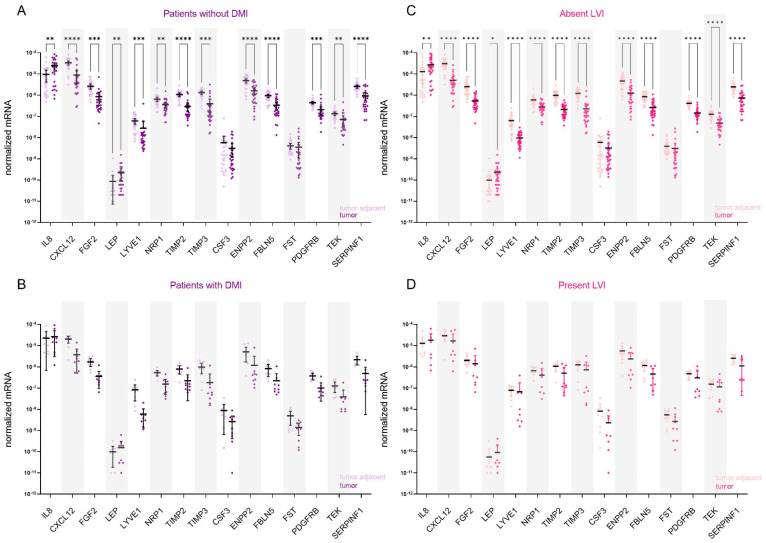
Expression of angiogenesis-related genes in EC patients stratified based on the presence of deep myometrial (DMI) or lymphovascular invasion (LVI). Expression of genes encoding 15 angiogenic factors in paired tissue samples from (**A**) patients without DMI (*n* = 27) and (**B**) from patients with present DMI (*n* = 9); tumour tissue is shown in dark purple and tumour-adjacent tissue in light purple colour. Expression of genes in paired tissue samples from (**C**) patients without LVI (*n* = 28) and (**D**) from patients with present LVI (*n* = 8). Tumour tissue is shown in dark pink, and the adjacent tissue is in light pink. Wilcoxon matched pairs signed rank tests with Bonferroni–Šidák corrections for multiple comparisons. Data are shown as scattered dot plots with marked means with 95% CI, * *p*-value ≤ 0.05, ** *p*-value ≤ 0.01, *** *p*-value ≤ 0.001, **** *p*-value ≤ 0.0001.

The stratification of data according to the presence or absence of deep myometrial or lymphovascular invasion revealed that genes for AFs are differentially expressed only in endometrial tissue from patients without DMI (*n* = 27; Figure 4A) or LVI (*n* = 28; Figure 4C). In both analyses, all genes except CSF3 and FST were differentially expressed between the tumour and tumour-adjacent tissue. In both patient categories, IL8 and LEP were up-regulated in tumour tissue: in patients without DMI, 5.8-fold and 5.4-fold, respectively, and in patients with absent LVI, 5.5-fold and 5.0-fold, respectively. Other genes were down-regulated in tumour versus adjacent tissue, most prominently CXCL12 (20.6-fold in patients without DMI and 21.7-fold in patients without LVI), followed by TIMP3 (15.0-fold in DMI absent and 16.2-fold in LVI-absent EC). No significant gene expression difference was detected in EC patients with DMI or LVI. 

Noteworthy, the groups of patients with FIGO IB-IV, high-grade EC, patients with LVI, and patients with DMI included in the study were small; thus, data obtained in these groups must be considered cautiously, and additional studies in larger groups of patients are needed.

##### There Is Much Broader Angiogenesis-Related Gene Involvement in Postmenopausal Women with EC than in Women of Reproductive Age 

**Figure 5:**
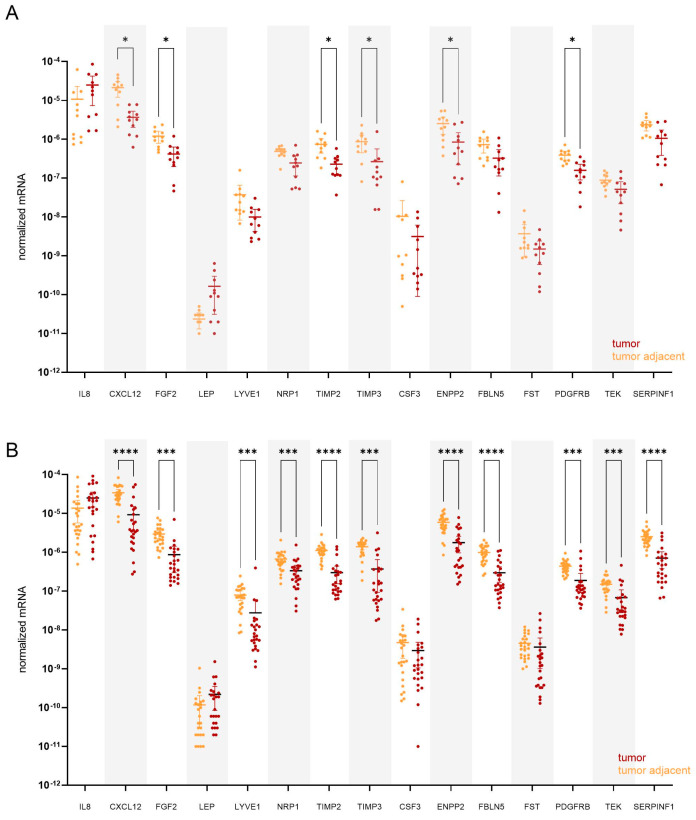
Expression of angiogenesis-related genes in paired tissue samples from (**A**) premenopausal (*n* = 11) and from (**B**) postmenopausal EC patients (*n* = 25). Tumour tissue is shown in dark red, and tumour-adjacent tissue is in orange colour. Wilcoxon matched pairs signed rank tests with Bonferroni–Šidák corrections for multiple comparisons. Data are shown as scattered dot plots with marked means with 95% CI, * *p*-value ≤ 0.05, *** *p*-value ≤ 0.001, **** *p*-value ≤ 0.0001.

Next, we stratified patients according to their menopausal status. In premenopausal patients (*n* = 11; Figure 5A), six genes were statistically significantly down-regulated in tumour tissue compared to tumour-adjacent tissue: TIMP3 (13.1-fold), ENPP2 (11.4-fold), FGF2 (7.6-fold), CXCL12 (7.5-fold), TIMP2 (5.2-fold), and PDGFRB (4.9-fold change). On the other hand, eleven genes were significantly down-regulated in postmenopausal women (*n* = 25; Figure 5B), most prominently CXCL12 (22.9-fold) and TIMP3 (15.5-fold change).

Finally, we separately compared the gene expression within the tumour and tumour-adjacent tissue in EC patients stratified according to EC grade, FIGO stage, presence of DMI, LVI, and menopausal status. ENPP2 was 2.4-fold down-regulated in tumour tissue, and *LYVE1* was 2.8-fold down-regulated in high-grade cancer compared to low-grade cancer. On the other hand, ENPP2 was 2.3-fold up-regulated within tumour-adjacent tissue, and *FGF2* was 2.4-fold up-regulated in postmenopausal women compared to premenopausal women. There was no statistically significant difference within the tumour tissue, nor in the tumour-adjacent tissue between patients with different FIGO stage EC, nor between the patients with a presence or absence of LVI or DMI (Figure S1).

#### 3.2.4. Co-Expression Patterns of the Genes: Higher Number of Strong Correlations Was Identified in EC Patients with Present LVI

Correlations between gene expressions were performed to highlight trends and relationships in the expression profiles of selected angiogenesis-related genes in T and TA tissues. Correlations between gene expressions in different tissues and inter-tissue expression relations were analysed. 

**Figure 6:**
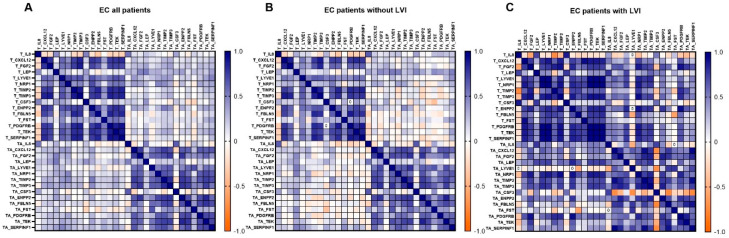
Gene co-expression pattern in tumour tissue, tumour-adjacent tissue, and between both of them. (**A**) All EC patients; *n* = 36, (**B**) EC patients without LVI; *n* = 28, (**C**) EC patients with LVI; *n* = 8. A Heatmap of Spearman correlation coefficients is shown. For details on strong correlations, see Table 5.

When all EC patients were considered, we identified five strong positive correlations with r > 0.85 and *p* < 0.05 within T tissue, with the strongest being between *PDGFRB* and *SERPINF1.* A list of all strong correlations is shown in Table 5. The pattern of gene correlations was similar within TA tissue; however, no correlations were considered strong (r > 0.85). No correlation between T and TA tissue gene expression reached r > 0.85 (Figure 6A). 

Next, patients were stratified according to LVI status. We identified similar correlation patterns between genes in EC patients without LVI to those in a group of all EC patients (Figure 6B). In patients without LVI, the strongest correlation found within T tissue was between *PDGFRB* and *SERPINF1* and within TA tissue between *ENPP2* and *TEK*.

A drastically higher number of strong correlations was identified in EC patients with present LVI. Within T tissue, 24 strong correlations with r > 0.85 were detected; the strongest correlations were between *PDGFRB* and *TEK*, between *LYVE1* and the genes *NRP1*, *PDGFRB*, and *TEK*, and between *SERPINF1* and the genes *NRP1*, *PDGFRB, TEK*, and *LYVE1*. Nine strong positive correlations were identified within TA tissue, the strongest between *CXCL12* and the genes *SERPINF1* and *TIMP2*, between *TIMP2* and the genes *SERPINF1* and *TIMP3*, and between *FBLN5* and *SERPINF1*. *CSF3* was negatively correlated with the majority of the other genes, but no relationship reached r > 0.85. 

Remarkably, when LVI was present, there was also a significant correlation between T versus TA tissue gene expression. We identified eight strong correlations between *NRP1* in TA and the following genes in T: *TIMP3*, *ENPP2*, *FGF2*, *NRP1*, *PDGFRB* and *TEK*; and between *PDGFRB* in TA and the following genes in T: *ENPP2* and *FGF2*. However, these results should be considered cautiously since the number of patients with LVI was low (*n* = 8).

### 3.3. Machine Learning Modelling Succeeded in Creating a Relatively Robust EC-Grade Prediction Model Based on the Tumour Gene Expressions 

#### 3.3.1. Data Normalisation Results

The results of the gamma distribution fitting (α, β) and the corresponding correlation coefficients for individual variables for the TCGA are shown in Table S4, and the results of the distribution fitting (α, β) and the corresponding correlation coefficients for the study dataset are shown in Table S5.

#### 3.3.2. Comparison of Training and Test Datasets

The training dataset contained 44 patients (75.9%), the testing dataset contained 14 patients (24.1%), and none of the 62 variables used in modelling significantly differed between the training and test datasets. Each variable’s Wilcoxon rank-sum statistic results are presented in Table S6. 

The training dataset contained 22 records (50%) from the TCGA dataset, and 22 records from the study dataset (50%), and the test dataset contained 14 records (100%) from the study dataset. Twenty-two records (50%) represented low-grade EC in the training dataset, and ten represented low-grade EC in the test dataset (71.4%).

#### 3.3.3. Modelling Results

**Figure 7:**
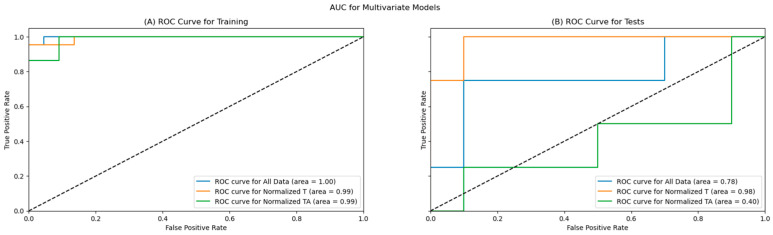
ROC Curves for the training (**A**) and test (**B**) datasets.

Of the three created models, all models showed a good prediction of the EC tumour grade (low/high) on the training dataset (AUC > 0.9), and two of the models kept good prediction capabilities on the holdout (test) data, including one of the models maintaining an AUC above 0.9. The ROC curves are presented in Figure 7, with the left (7A) figure representing the ROC curves for the training dataset and the right (7B) figure showing the ROC curves for the test dataset.

As shown in Table 6, all primary metrics for the EC tumour grade classification for all three models are high on the training data, with the accuracy, precision, F1, and specificity for all three models reaching or exceeding 90%, with AUC scores near 1.00. The remaining metrics exceeded 80% for all models on the training dataset.

The primary metrics remained high on two models for EC tumour grade prediction; specifically, the models utilising the data from the tumour tissue (all data and normalised tumour data), as shown in Table 7. The best-performing model on the test dataset is based only on tumour data, reaching an accuracy above 85%, with 100% recall and sensitivity, 80% specificity, and 66.7% precision; the AUC of the model remained very high at 0.98. The second model that performed well was the model using all data, which also reached an accuracy above 85%, with a slightly better precision at 75%, but reduced recall (75%) and F1 score, and a lower AUC score of 0.78. The model utilising only the tumour-adjacent tissue performed similarly to a random model, with an accuracy of 50%.

**Figure 8:**
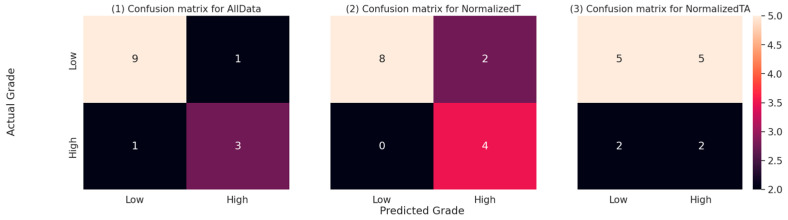
Confusion matrices for all three models on the test dataset. The numbers represent the number of predictions falling into each of the combinations of actual (real) EC tumour grade and the EC tumour grade predicted by the respective model. The cell colours represent the relative numbers, with darker colours representing lower counts and lighter colours representing higher counts.

The confusion matrices for the EC tumour grade prediction on the test dataset, which form the basis for the metrics in Table 7, are presented in Figure 8 below. According to Fisher’s exact statistics, the confusion matrices for all data and normalised tumour models are statistically significant (all data: *p* < 0.05; tumour: *p* < 0.05). The confusion matrix for the model using the adjacent tissue data is not significant.

## 4. Discussion

In this study, we evaluated the expression of 15 genes known for their involvement in angiogenic processes, 9 of which were selected from publicly available libraries, TCGA [19,20,21] and CPTAC UCEC [22], out of 91 different angiogenesis-related genes and 64 encoded proteins, respectively, based on their differential detection in EC T tissue in comparison to morphologically normal adjacent endometrial tissue. Those nine genes were the following: *CXCL12*, *ENPP2*, *FBLN5*, *FGF2*, *LYVE1*, *PDGFRB*, *SERPINF1*, *TIMP2*, and *TIMP3*. Six genes, *CSF3*, *IL8*, *LEP*, *NRP1*, *TEK*, and *FST*, were preselected in our previous research [14,18] on plasma samples from EC patients. To the best of our knowledge, this is the largest set of genes involved in angiogenesis analysed in EC tissue. 

Using a clinical cohort of 36 EC patients, we confirmed the difference between T and TA tissue expression in EC for thirteen out of fifteen analysed genes involved in angiogenesis. According to TCGA data, all nine genes selected from data libraries are down-regulated in T versus TA tissue, with an expression ratio range from −17.5 (*FGF2*) to −3.5 (*PDGFRB)*. We observed similar T-to-TA expression ratios for individual genes in the clinical cohort. Out of six additional genes, *LEP* and *IL8* were significantly up-regulated, and *TEK* was significantly down-regulated in T tissue, which is in accordance with our previous research, where plasma levels of EC patients were compared to those of control patients with benign gynaecological pathology [14,18]. In contrast to our plasma research, in the present tissue-based study, *NRP1* was significantly down-regulated, and the differences in T versus TA expression for *CSF3* and *FST* were insignificant. 

Interestingly, all the genes selected from TCGA were down-regulated in T tissue compared to TA tissue (Figure 1A), highlighting the importance of the antiangiogenic lever of the angiogenic switch in the endometrial tissue. The endometrial tissue’s uniqueness is its cyclic exposure to extensive hormonal changes. During each monthly cycle, ovarian hormones trigger angiogenic processes and endometrium regeneration, followed by blood vessel loss. Endometrial tissue thus produces both pro- and anti-AFs [11,37]. The levels of various main AF groups, like angiopoietins, VEGFs, and MMPs, fluctuate during different menstrual cycle phases [38,39]. EC, however, occurs mainly in postmenopausal women where the expression of AF is not exposed to altered concentrations of ovarian hormones. When screening TCGA library data in EC, genes encoding these AFs were not differentially expressed between T and TA tissue, while on the other hand, genes for their endogenous inhibitors, i.e., *TEK* (binding to angiopoietins), *NRP1*, *PDGFRB*, *FBLN5*, *SERPINF1* (receptors and inhibitors of VEGF), *TIMP2*, and *TIMP3* (inhibitors of MMPs) were substantially down-regulated in T versus TA tissue. While pro-angiogenic factors promote angiogenesis during the normal monthly menstrual cycle [38], our data suggest that tumour angiogenesis in EC is promoted mainly by the decreased gene expression of various antiangiogenic factors. 

In our study, *CXCL12* was the most down-regulated gene in all analysed strata: it was 18.2-fold down-regulated in all EC patients and 20.9-fold, 21.9-fold, 20.6-fold, and 21.7-fold down-regulated, respectively, in low-grade EC, in low stage EC, in DMI-absent EC, and LVI-absent EC patients. CXCL12 is a chemokine that plays a critical role as a chemoattractant in the tumour niche. Its secretion by myofibroblasts stimulates tumour progression [40]. The CXCL12/CXCR4 axis plays a vital role in endometrial cancer’s proliferation, invasion, and metastasis [41]. CXCL12 primarily binds to its receptor CXCR4 to regulate the trafficking of both normal and malignant cells. In a paracrine manner, CXCL12 attracts CXCR4-expressing tumour cells to a new tumour niche, resulting in tumour cell invasion and metastasis [42,43]. CXCL12/CXCR4 also has essential roles in the muscular infiltration of endometrial cancer by activating the PI3K/Akt signalling pathway [44]. Different authors have reported that CXCL12/CXCR4 expression in human EC tissues was inversely related to the histological grade, whereas survival rates were significantly better in patients with higher levels of CXCR4 [45]. 

EC is a heterogeneous disease; different angiogenic mechanisms are expressed during different endometrial cancer phases. We stratified patients according to their clinical and histopathological characteristics. Similar observations were reached in all analyses. In patients with prognostically more favourable forms of EC—in less advanced stages and lower grades of EC, genes for AFs tended to be differentially expressed in T tissue compared to non-cancerous TA tissue. On the contrary, differences in gene expressions are less prominent in more advanced or aggressive forms of EC. Likewise, stratification according to the presence or absence of deep myometrial or lymphovascular invasion identified differentially expressed genes only in endometrial tissue from patients without DMI or LVI, whereas no significant difference was detected in gene expression in EC patients with DMI or LVI. The groups of patients with FIGO IB-IV, high-grade EC, patients with LVI, and patients with DMI included in our study were small; thus, data obtained in these groups must be considered cautiously, and additional studies in larger groups of patients are needed.

Nevertheless, the finding concurs with published data on various genes coding for AFs. The loss of TIMP3 correlated with advanced-stage disease and poor prognosis in various cancers [46]. A decreasing expression of *TIMP-2* in EC tissue was correlated with the histological grade of EC, with the level of myometrial invasion, lymphovascular space involvement, and lymph node involvement [47]. 

We also examined the co-expressions of analysed genes within T and TA tissue in EC patients stratified according to LVI status, the cornerstone of risk stratification in EC [7]. Curiously, we identified a drastically higher number of strongly correlated mRNA expressions in both T and TA tissue when LVI was present. There was also a significant correlation between eight gene expressions in T versus TA tissue, which was not observed in the absence of LVI. Primarily, in T tissue and in LVI-positive samples, numerous strong correlations were found between the expression of *SERPINF*1 and other angiogenesis-related genes, which has not been acknowledged before in the STRING database. Due to the low number of samples with LVI, these findings should be additionally analysed in a larger cohort. SERPINF1 is a known antiangiogenic factor with many additional functions like anti-tumour, anti-inflammation, nutrition, and nerve protection functions, and is involved in fat metabolism. In various cancers, including EC, the expression of SERPINF1 is lower in tumour tissue than in control tissue [48]. It is expressed in the normal and cancerous endometrium, and its loss of expression is associated with endometrial hyperplasia, a precursor for EC, and increased EC proliferation [49]. 

Next, we stratified EC patients by menopausal status. Six genes were significantly down-regulated in the reproductive age group and eleven in postmenopausal women. The diversity of genes supporting angiogenesis in EC in younger populations was much lower than in postmenopausal patients. All the down-regulated genes in premenopausal patients were, with even higher significance, down-regulated in postmenopausal patients, while six additional genes were down-regulated in the latter group. This indicates broader angiogenesis-related gene involvement in postmenopausal women with EC, which is in accordance with findings of an age- and menopausal-status-related increase in somatic mutation frequency across many tumour types [50,51]. One of the genes with the most reduced expression in both menopausal status subgroups was tissue inhibitor of metalloproteinases, TIMP3, the silencing of which is consistently associated with cancer progression or poor patient prognosis in multiple human cancers, including EC. TIMP3 promoter is a frequently targeted methylation site, and its epigenetic silencing indicates a pro-tumorigenic outcome [46,52]. 

EC often involves patients with other comorbidities, like obesity, an established risk factor for EC [53,54,55,56,57]. Leptin, an adipokine encoded by the *LEP* gene, has an important role in energy balance and glucose metabolism. It plays an integral part in the link between obesity and EC, where the tyrosine kinase-dependent intracellular pathway promotes angiogenesis during cancer development [58,59,60]. There is a continuous debate with contradicting results on whether the effect of leptin on EC risk is related to higher BMI or whether it is an independent risk factor for EC [61,62,63]. In our previous research, we used an automated machine learning approach through which we showed that in univariate and multivariate models, leptin might predict EC better than BMI [14]. This supports findings that leptin might be involved in EC development through mechanisms beyond obesity-related pathophysiology, including through angiogenesis [59,60]. The overexpression of *LEP* in endometrioid EC compared to benign control patients was reported before [64]. Indeed, in our study, the expression of the *LEP* gene was significantly elevated in tumour tissue compared to patient-paired, morphologically normal, adjacent endometrial tissue (mean FR = 4.68; 95% CI 2.87–6.49; *p* = 0.0104; Table 4), which directly supports the hypothesis of leptin’s independent role in EC carcinogenesis. 

Besides leptin, IL8 mRNA expression was up-regulated in T versus TA tissue (mean FR = 4.75; 95% CI 2.46–7.04; *p* = 0.0164), which supports our previous findings in plasma [14]. IL-8 is a pro-inflammatory cytokine secreted by adipocytes and represents another link between adipose tissue and EC [65,66]. It is chemotactic for lymphocytes and neutrophils and has an important role in angiogenesis [67,68]. Elevated IL-8 serum levels were independently associated with shorter disease-free and overall survival in EC cancer [69]. The protein–protein interaction analysis using the STRING database [33] revealed strong interaction and gene co-expression between *IL8* and *CSF3* (Figure 1D). Our data confirmed this interaction, but only within T tissue in EC patients with LVI present (*r* = 0.881; *p* = 7.2 × 10^−3^; Table 5). 

Fertility-sparing treatment is considered for endometrioid patients with endometrial carcinoma of a low grade [8]. In order to verify whether the selected AF levels in the tumour or adjacent tissue could be used for EC grade stratification, we developed three different models utilising automated machine learning approaches using the differentially expressed genes identified on the TCGA dataset. While all models received near-perfect scores on the training dataset, the model based on the TA data did not generalise well (achieved random scores on the test data), which was probably caused by overfitting, as the sample was very small. However, the model based on the T tissue data retained excellent scores on the test data, retaining AUC at the 0.9 level, with a sensitivity of 100% and specificity of 80%. Caution is always required when interpreting results on such small datasets, but further multicentre validation studies, including one specifically for reproductive-age women, would probably be warranted, given the statistical significance of the result on the test dataset.

Our data indicate that the regulation of angiogenesis-related genes in EC with prognostically less favourable characteristics is not limited to T tissue alone but rather spreads onto the non-cancerous TA tissue of the surrounding endometrium. This is in accordance with the fact that TA tissues are often involved in the development and progression of the tumour [70,71,72]. TA tissue is a distinct tissue type that presents a unique intermediate state between healthy tissue and tumour tissue [72]. Several studies suggest that TA tissue may offer helpful information for predicting disease prognosis [73]. This may be due to either (1) field cancerisation theory, which suggests that paired TA tissues are in an intermediate state between normal and tumour, thus bearing information on early tumour initialisation and development, or (2) tumour microenvironment theory, which suggests that TA tissues contain information about the microenvironment surrounding tumours, which either promotes or suppresses tumour development [73]. There is increasing evidence that a single genetic mutation is insufficient to initiate the disease and that microenvironment-derived signals may be required to drive tumour progression. The neoplastic and non-neoplastic cells in the microenvironment communicate to produce a microenvironment favourable for the progression of endometrial carcinogenesis [43,45]. According to different lines of evidence, genomic data from non-cancerous TA tissue can independently predict cancer survival, and, in some cases, provide even superior performance relative to models based on tumour-derived data alone [71,73,74]. We found fewer differences between angiogenesis-related gene expression in T versus TA tissue in higher stages and grades of EC, indicating that the progression of the tumour was not only related to the expression of AFs in the tumorous tissue but also to the expression in the TA tissue samples. While our machine learning modelling did not confirm the ability to use TA tissue for cancer grade classification, it is worth stressing that the models for TA did overfit due to sample size limitations, and it is therefore impossible to draw direct conclusions from the result.

Additionally, in order to be able to model the combined data from the TCGA dataset and the data from our study, a novel approach to data merging (and normalisation) was utilised, building upon previous ideas on combining different datasets [25,28]. As the results are promising, a further study to identify whether the method could be generalised might be interesting.

It is again important to stress that the overall sample size used in the study is a limitation and results should be interpreted with that in mind. This could be addressed in the future by conducting a study in a larger group of patients, ideally in multiple geographically dispersed centres using the same clinical protocol. 

## 5. Conclusions

We showed that angiogenesis in EC is promoted mainly by the decreased gene expression of antiangiogenic factors. Our data also indicate that the regulation of angiogenesis-related genes in EC with prognostically less favourable characteristics, i.e., higher cancer stage or grade, or the presence of LVI or DMI, is affected not only in T but also in the TA tissue of the surrounding endometrium, where gene expression is altered inside morphologically normal cells within the tumour microenvironment. However, we identified stronger gene co-expressions in T than in TA tissue; correlations were particularly strong when the lymphovascular invasion was present. We also confirmed broader AF gene involvement in postmenopausal compared to premenopausal women with EC. Additionally, by combining TCGA data and data from our study, we applied machine learning modelling to create a relatively robust model, able to differentiate between low-grade and high-grade EC based on the T gene expressions, which might be helpful in fertility-sparing settings in EC patients.

## Figures and Tables

**Table 1:** List of publicly available datasets used in this study.

Dataset	Downloaded from	Detail	Samples	References
cBioPortal	https://www.cbioportal.org/ accessed on 4 July 2022	TCGA Pan-Cancer study	*n* (T) = up to 527	[19,20]
UCSC Xena	https://xena.ucsc.edu/ accessed on 8 July 2022	GDC TCGA Endometrioid Cancer (UCEC) study (TCGA data uniformly reanalysed at GDC by UCSC Xena group using the latest Human Genome Assembly hg38)	*n* (T) = up to 548; *n* (TA) = up to 35; *n* (paired samples) = up to 23.	[21]
NCI PDC server	https://proteomic.datacommons.cancer.gov/pdc/ accessed on 5 July 2022	CPTAC UCEC Discovery Study—Proteome, PDC ID: PDC000125, study ID: c935c587-0cd1-11e9-a064-0a9c39d33490	*n* (T) = up to 95; *n* (TA) = up to 25; *n* (paired samples) = up to 24.	[22]

TCGA—The Cancer Genome Atlas; CPTAC—Clinical Proteomic Tumor Analysis Consortium; UCEC—Uterine Corpus Endometrial Carcinoma.

**Table 2:** Detailed information about the patients included in the study.

Sample	Age	Menopausal Status	Histological Type/Grade	FIGO Stage	Gradus HG/LG	Depth of Myometrial Invasion	Lymphovascular Invasion
5	39	premenopausal	dedifferentiated	IB	HG	>50%	yes
7	50	premenopausal	endometrioid G1	IB	LG	no	no
8	83	postmenopausal	dedifferentiated	IB	HG	>50%	no
9	41	premenopausal	endometrioid G1	IA	LG	<50%	no
10	53	postmenopausal	endometrioid G1	IA	LG	no	no
13	64	postmenopausal	endometrioid G1	IV	LG	<50%	NA
14	73	postmenopausal	endometrioid G1	IB	LG	>50%	no
16	69	postmenopausal	endometrioid G1	IA	LG	<50%	no
18	79	postmenopausal	endometrioid G1	IB	LG	>50%	no
19	74	postmenopausal	endometrioid G1	IA	LG	<50%	no
20	76	postmenopausal	endometrioid G1	IA	LG	<50%	no
21	53	premenopausal	endometrioid G2	IA	LG	no	no
23	45	premenopausal	endometrioid G1	IA	LG	no	no
24	69	postmenopausal	endometrioid G2	IB	LG	>50%	yes
25	54	premenopausal	endometrioid G3	IA	HG	<50%	no
26	72	postmenopausal	endometrioid G1	IA	LG	<50%	no
30	54	premenopausal	endometrioid G1	IA	LG	no	no
33	77	postmenopausal	endometrioid G3	IB	HG	>50%	no
34	57	postmenopausal	mucinous	IA	LG	<50%	no
40	71	postmenopausal	serous	IA	HG	<50%	no
44	73	postmenopausal	serous	IB	HG	>50%	yes
47	27	premenopausal	dedifferentiated	IA	HG	<50%	no
49	70	postmenopausal	endometrioid G1	IA	LG	<50%	no
50	73	postmenopausal	endometrioid G1	IA	LG	<50%	no
51	75	postmenopausal	endometrioid G2	IA	LG	>50%	yes
52	75	postmenopausal	endometrioid G2	IA	LG	<50%	yes
53	50	postmenopausal	endometrioid G3	IA	HG	<50%	yes
54	71	postmenopausal	endometrioid G1	IA	LG	<50%	no
56	55	postmenopausal	endometrioid G1	IA	LG	no	no
57	43	premenopausal	endometrioid G1	IA	LG	no	no
62	59	postmenopausal	endometrioid G1	IA	LG	no	no
63	66	postmenopausal	endometrioid G1	IA	LG	<50%	no
65	80	postmenopausal	carcinosarcoma	IB	HG	>50%	yes
66	72	postmenopausal	endometrioid G1	IA	LG	<50%	no
68	45	premenopausal	endometrioid G1	II	LG	<50%	no
71	48	premenopausal	serous	IA	HG	<50%	no

G—gradus, HG—high grade, LG—low grade.

**Table 3:** Details for the TaqMan “Assays on Demand” used for the 15 investigated genes and 2 reference genes.

Gene Symbol	Gene/AF Name	Assay ID
*CSF3*	colony stimulating factor 3	Hs99999083_m1
*CXCL12*	C-X-C motif chemokine ligand 12	Hs00171022_m1
*ENPP2*	ectonucleotide pyrophosphatase/phosphodiesterase 2	Hs00196470_m1
*FBLN5*	fibulin 5	Hs00197064_m1
*FGF2*	fibroblast growth factor 2	Hs00266645_m1
*FST*	follistatin	Hs00246256_m1
HPRT1 *	hypoxanthine-guanine phosphoribosyltransferase	Hs02758991_g1
*IL8*	C-X-C motif chemokine ligand 8	Hs00174103_m1
*LEP*	leptin	Hs00174877_m1
*LYVE1*	lymphatic vessel endothelial hyaluronan receptor 1	Hs00272659_m1
*NRP1*	neuropilin 1	Hs00826128_m1
*PDGFRB*	platelet derived growth factor receptor beta	Hs00387364_m1
POLR2A *	DNA-directed RNA polymerase II subunit RPB1	Hs00426592_m1
*SERPINF1*	serpin family F member 1	Hs00171467_m1
*TEK*	TEK receptor tyrosine kinase; Tie-2	Hs00176096_m1
*TIMP2*	TIMP metallopeptidase inhibitor 2	Hs00234278_m1
*TIMP3*	TIMP metallopeptidase inhibitor 3	Hs00165949_m1

* Reference gene.

**Table 4:** Comparison of fold regulation (FR) in gene expression between tumour and tumour-adjacent control tissue.

Genes	Mean FR	FR CI 95%		*p*-Value ^‡^	
*IL8*	4.75	2.46	7.04	0.0164	*
*CXCL12*	−18.21	−31.95	−4.46	0.0015	**
*FGF2*	−7.39	−10.51	−4.27	0.0015	**
*LEP*	4.68	2.87	6.49	0.0104	*
*LYVE1*	−10.98	−16.52	−5.44	0.0015	**
*NRP1*	−3.67	−5.11	−2.22	0.0015	**
*TIMP2*	−6.79	−8.87	−4.70	0.0015	**
*TIMP3*	−14.77	−21.77	−7.77	0.0015	**
*CSF3*	−10.43	−20.51	−0.35	0.8328	ns
*ENPP2*	−10.10	−15.05	−5.14	0.0015	**
*FBLN5*	−8.27	−12.70	−3.83	0.0015	**
*FST*	−6.77	−9.98	−3.56	0.0668	ns
*PDGFRB*	−4.50	−6.08	−2.92	0.0015	**
*TEK*	−5.20	−7.50	−2.91	0.0015	**
*SERPINF1*	−8.68	−12.71	−4.66	0.0015	**

^‡^ adjusted *p*-values after Wilcoxon matched-pairs signed rank test with Bonferroni–Šidák corrections for multiple comparisons. * *p*-value ≤ 0.05, ** *p*-value ≤ 0.01, ns—not significant.

**Table 5:** Strong correlations between mRNA expressions within the tumour and tumour-adjacent tissue and between both tissues, considering EC patients stratified according to LVI status.

	Tumour Tissue				Tumour-Adjacent Tissue				Tumour Tissue vs. Tumour-Adjacent Tissue			
	Gene_T	Gene_T	r	*p*-Value	Gene_TA	Gene_TA	r	*p*-Value	Gene_T	Gene_TA	r	*p*-Value
All EC Patients	*PDGFRB*	*SERPINF1*	0.906	3.3 × 10^−14^								
	*TEK*	*SERPINF1*	0.888	4.9 × 10^−13^								
	*TIMP2*	*TIMP3*	0.879	1.7 × 10^−12^								
	*FGF2*	*TIMP3*	0.871	4.9 × 10^−12^								
	*PDGFRB*	*TEK*	0.866	9.2 × 10^−12^								
EC Patients without LVI	*PDGFRB*	*SERPINF1*	0.878	7.9 × 10^−10^	*ENPP2*	*TEK*	0.854	7.2 × 10^−9^				
	*CXCL12*	*TIMP3*	0.875	1.1 × 10^−9^								
	*FBLN5*	*PDGFRB*	0.873	1.4 × 10^−9^								
	*TIMP2*	*SERPINF1*	0.858	5.3 × 10^−9^								
	*TIMP2*	*TIMP3*	0.857	5.8 × 10^−9^								
EC Patients with LVI	*PDGFRB*	*TEK*	1.000	4.9 × 10^−5^	*CXCL12*	*SERPINF1*	0.952	0.001	*TIMP3*	*NRP1*	0.929	0.002
	*LYVE1*	*NRP1*	0.976	4.0 × 10^−4^	*CXCL12*	*TIMP2*	0.929	0.002	*ENPP2*	*PDGFRB*	0.929	0.002
	*LYVE1*	*PDGFRB*	0.976	4.0 × 10^−4^	*TIMP2*	*SERPINF1*	0.929	0.002	*FGF2*	*PDGFRB*	0.905	0.005
	*LYVE1*	*TEK*	0.976	4.0 × 10^−4^	*TIMP2*	*TIMP3*	0.905	0.005	*ENPP2*	*NRP1*	0.905	0.005
	*NRP1*	*SERPINF1*	0.976	4.0 × 10^−4^	*FBLN5*	*SERPINF1*	0.905	0.005	*FGF2*	*NRP1*	0.881	0.007
	*PDGFRB*	*SERPINF1*	0.976	4.0 × 10^−4^	*FGF2*	*TIMP3*	0.857	0.011	*NRP1*	*NRP1*	0.881	0.007
	*TEK*	*SERPINF1*	0.976	4.0 × 10^−4^	*FGF2*	*ENPP2*	0.857	0.011	*PDGFRB*	*NRP1*	0.881	0.007
	*LYVE1*	*SERPINF1*	0.952	1.1 × 10^−3^	*TIMP3*	*ENPP2*	0.857	0.011	*TEK*	*NRP1*	0.881	0.007
	*CXCL12*	*FST*	0.922	2.6 × 10^−3^	*PDGFRB*	*TEK*	0.857	0.011				
	*FGF2*	*TIMP3*	0.905	4.6 × 10^−3^								
	*NRP1*	*TIMP3*	0.905	4.6 × 10^−3^								
	*TIMP3*	*PDGFRB*	0.905	4.6 × 10^−3^								
	*TIMP3*	*TEK*	0.905	4.6 × 10^−3^								
	*IL8*	*CSF3*	0.881	7.2 × 10^−3^								
	*CXCL12*	*NRP1*	0.881	7.2 × 10^−3^								
	*CXCL12*	*PDGFRB*	0.881	7.2 × 10^−3^								
	*CXCL12*	*TEK*	0.881	7.2 × 10^−3^								
	*TIMP3*	*ENPP2*	0.881	7.2 × 10^−3^								
	*TIMP3*	*SERPINF1*	0.881	7.2 × 10^−3^								
	*CXCL12*	*TIMP3*	0.857	1.1 × 10^−2^								
	*CXCL12*	*SERPINF1*	0.857	1.1 × 10^−2^								
	*FGF2*	*TIMP2*	0.857	1.1 × 10^−2^								
	*LYVE1*	*TIMP3*	0.857	1.1 × 10^−2^								
	*ENPP2*	*SERPINF1*	0.857	1.1 × 10^−2^								

r—Spearman correlation coefficient, *p* < 0.05 is considered significant.

**Table 6:** Primary metrics achieved on the training dataset.

Model	Accuracy	Precision	Recall	F1	AUC	Sensitivity	Specificity
All Data	93.2%	100%	86.4%	92.7%	1.00	86%	100%
Tumour (Normalised T)	90.9%	100%	81.8%	90%	0.99	82%	100%
Adjacent (Normalised TA)	95.5%	91.7%	100%	95.7%	0.99	100%	91%

**Table 7:** Primary metrics achieved on the test dataset.

Model	Accuracy	Precision	Recall	F1	AUC	Sensitivity	Specificity
All Data	85.7%	75%	75%	75%	0.78	75%	90%
Tumour (Normalised T)	85.7%	66.7%	100%	80%	0.98	100%	80%
Adjacent (Normalised TA)	50%	28.6%	50%	36.4%	0.40	50%	50%

## Data Availability

The datasets used and/or analysed during the current study are available from the corresponding authors on reasonable request. Machine learning modelling: the complete MATLAB, Jupyter Notebook, and Python scripts are available here: https://github.com/klokedm/EndometrialCancerGradePrediction.

## Supplementary Materials

The following supporting information can be downloaded at https://www.mdpi.com/article/10.3390/cancers15143661/s1, Table S1: Normalized expression of angiogenesis-associated genes in endometrial cancer tissues and adjacent control tissues; data from the GDC TCGA Endometrioid Cancer (UCEC) study; Table S2: Normalized levels of angiogenesis-associated proteins in endometrial cancer tissues and adjacent control tissues; data from the CPTAC UCEC Discovery Study—Proteome; Table S3: Expression of angiogenesis-associated genes in endometrial cancer tissue (*n* = up to 527). Data are from the TCGA Pan-Cancer study; Table S4: The calculated distribution fit correlation with the original data, including the *p*-values and estimated upper and lower bounds for the 95% confidence intervals for the TCGA dataset; Table S5: The calculated distribution fit correlation with the original data, including the *p*-values and estimated upper and lower bounds for the 95% confidence intervals for the study dataset; Table S6: Comparison between the training and test datasets using the Wilcoxon rank-sum Test; Figure S1: Volcano plots identifying significant changes in gene expression within the tumour and tumour-adjacent tissue separately, in EC patients stratified according to the presence of LVI or DMI, EC grade, FIGO stage, and menopausal status.

## References

[1] Crosbie E.J., Kitson S.J., McAlpine J.N., Mukhopadhyay A., Powell M.E., Singh N. (2022). Endometrial cancer. Lancet.

[2] Zhang S., Gong T.T., Liu F.H., Jiang Y.T., Sun H., Ma X.X., Zhao Y.H., Wu Q.J. (2019). Global, Regional, and National Burden of Endometrial Cancer, 1990–2017: Results from the Global Burden of Disease Study, 2017. Front. Oncol..

[3] Lee N.K., Cheung M.K., Shin J.Y., Husain A., Teng N.N., Berek J.S., Kapp D.S., Osann K., Chan J.K. (2007). Prognostic factors for uterine cancer in reproductive-aged women. Obstet. Gynecol..

[4] Wilczyński M., Danielska J., Wilczyński J. (2016). An update of the classical Bokhman’s dualistic model of endometrial cancer. Przegląd Menopauzalny.

[5] Murali R., Soslow R.A., Weigelt B. (2014). Classification of endometrial carcinoma: More than two types. Lancet Oncol..

[6] Kandoth C., Schultz N., Cherniack A.D., Akbani R., Liu Y., Shen H., Robertson A.G., Pashtan I., Shen R., Cancer Genome Atlas Research Network (2013). Integrated genomic characterization of endometrial carcinoma. Nature.

[7] Concin N., Matias-Guiu X., Vergote I., Cibula D., Mirza M.R., Marnitz S., Ledermann J., Bosse T., Chargari C., Fagotti A. (2021). ESGO/ESTRO/ESP guidelines for the management of patients with endometrial carcinoma. Int. J. Gynecol. Cancer.

[8] Rodolakis A., Scambia G., Planchamp F., Acien M., Di Spiezio Sardo A., Farrugia M., Grynberg M., Pakiž M., Pavlakis K., Vermeulen N. (2023). ESGO/ESHRE/ESGE Guidelines for the fertility-sparing treatment of patients with endometrial carcinoma. Int. J. Gynecol. Cancer.

[9] Helpman L., Kupets R., Covens A., Saad R.S., Khalifa M.A., Ismiil N., Ghorab Z., Dubé V., Nofech-Mozes S. (2014). Assessment of endometrial sampling as a predictor of final surgical pathology in endometrial cancer. Br. J. Cancer.

[10] Risau W. (1997). Mechanisms of angiogenesis. Nature.

[11] Abulafia O., Sherer D.M. (1999). Angiogenesis of the endometrium. Obstet. Gynecol..

[12] Carmeliet P., Jain R.K. (2000). Angiogenesis in cancer and other diseases. Nature.

[13] Roškar L., Roškar I., Rižner T.L., Smrkolj Š. (2021). Diagnostic and Therapeutic Values of Angiogenic Factors in Endometrial Cancer. Biomolecules.

[14] Roškar L., Pušić M., Roškar I., Kokol M., Pirš B., Smrkolj Š., Rižner T.L. (2022). Models including preoperative plasma levels of angiogenic factors, leptin and IL-8 as potential biomarkers of endometrial cancer. Front. Oncol..

[15] Karkia R., Wali S., Payne A., Karteris E., Chatterjee J. (2022). Diagnostic Accuracy of Liquid Biomarkers for the Non-Invasive Diagnosis of Endometrial Cancer: A Systematic Review and Meta-Analysis. Cancers.

[16] Obradović D.D., Milić N.M., Miladinović N., McClements L., Oprić D.M. (2022). Loss of Expression of Antiangiogenic Protein FKBPL in Endometrioid Endometrial Carcinoma: Implications for Clinical Practice. Medicina.

[17] Kokol P., Kokol M., Zagoranski S. (2022). Machine learning on small size samples: A synthetic knowledge synthesis. Sci. Prog..

[18] Roškar L., Klančič T., Knific T., Rižner T.L., Smrkolj Š. (2021). Tie-2, G-CSF, and Leptin as Promising Diagnostic Biomarkers for Endometrial Cancer: A Pilot Study. J. Clin. Med..

[19] Gao J., Aksoy B.A., Dogrusoz U., Dresdner G., Gross B., Sumer S.O., Sun Y., Jacobsen A., Sinha R., Larsson E. (2013). Integrative analysis of complex cancer genomics and clinical profiles using the cBioPortal. Sci. Signal.

[20] Weinstein J.N., Collisson E.A., Mills G.B., Mills Shaw K.R., Ozenberger B.A., Ellrott K., Shmulevich I., Sander C., Stuart J.M. (2013). The Cancer Genome Atlas Pan-Cancer analysis project. Nat. Genet..

[21] Goldman M.J., Craft B., Hastie M., Repečka K., McDade F., Kamath A., Banerjee A., Luo Y., Rogers D., Brooks A.N. (2020). Visualizing and interpreting cancer genomics data via the Xena platform. Nat. Biotechnol..

[22] Dou Y., Kawaler E.A., Cui Zhou D., Gritsenko M.A., Huang C., Blumenberg L., Karpova A., Petyuk V.A., Savage S.R., Satpathy S. (2020). Proteogenomic Characterization of Endometrial Carcinoma. Cell.

[23] Pavlič R., Vidic S., Anko M., Knific T., Büdefeld T., Marton K., Sinreih M., Poschner S., Jäger W., Frković-Grazio S. (2021). Altered Profile of E1-S Transporters in Endometrial Cancer: Lower Protein Levels of ABCG2 and OSTβ and Up-Regulation of SLCO1B3 Expression. Int. J. Mol. Sci..

[24] Bustin S.A., Benes V., Garson J.A., Hellemans J., Huggett J., Kubista M., Mueller R., Nolan T., Pfaffl M.W., Shipley G.L. (2009). The MIQE Guidelines: Minimum Information for Publication of Quantitative Real-Time PCR Experiments. Clin. Chem..

[25] Song K., Zhou Y.-H. (2023). Leveraging Scheme for Cross-Study Microbiome Machine Learning Prediction and Feature Evaluations. Bioengineering.

[26] Lee A.J., Park Y.S., Doing G., Hogan D.A., Greene C.S. (2020). Correcting for experiment-specific variability in expression compendia can remove underlying signals. GigaScience.

[27] Butler A., Hoffman P., Smibert P., Papalexi E., Satija R. (2018). Integrating single-cell transcriptomic data across different conditions, technologies, and species. Nat. Biotechnol..

[28] Friedman N., Cai L., Xie X.S. (2006). Linking stochastic dynamics to population distribution: An analytical framework of gene expression. Phys. Rev. Lett..

[29] Deisenroth M.P., Faisal A.A., Ong C.S. (2021). Mathematics for Machine Learning.

[30] Piońska A., Pioński P. (2021). MLJAR: State-of-the-art Automated Machine Learning Framework for Tabular Data. Version 0.10.3.

[31] Pedregosa F., Varoquaux G., Gramfort A., Michel V., Thirion B., Grisel O., Blondel M., Prettenhofer P., Weiss R., Dubourg V. (2011). Scikit-learn: Machine Learning in Python. J. Mach. Learn Res..

[32] The Pandas Development Team (2020). Pandas-Dev/Pandas: Pandas. Zenodo.

[33] Szklarczyk D., Gable A.L., Nastou K.C., Lyon D., Kirsch R., Pyysalo S., Doncheva N.T., Legeay M., Fang T., Bork P. (2021). The STRING database in 2021: Customizable protein–protein networks, and functional characterization of user-uploaded gene/measurement sets. Nucleic Acids Res..

[34] Amant F., Mirza M.R., Koskas M., Creutzberg C.L. (2018). Cancer of the corpus uteri. Int. J. Gynecol. Obstet..

[35] Voss M.A., Ganesan R., Ludeman L., McCarthy K., Gornall R., Schaller G., Wei W., Sundar S. (2012). Should grade 3 endometrioid endometrial carcinoma be considered a type 2 cancer-A clinical and pathological evaluation. Gynecol. Oncol..

[36] Setiawan V.W., Yang H.P., Pike M.C., McCann S.E., Yu H., Xiang Y.B., Wolk A., Wentzensen N., Weiss N.S., Webb P.M. (2013). Type I and II endometrial cancers: Have they different risk factors?. J. Clin. Oncol..

[37] Yetkin-Arik B., Kastelein A.W., Klaassen I., Jansen C.H.J.R., Latul Y.P., Vittori M., Biri A., Kahraman K., Griffioen A.W., Amant F. (2021). Angiogenesis in gynecological cancers and the options for anti-angiogenesis therapy. Biochim. Biophys. Acta Rev. Cancer.

[38] Demir R., Yaba A., Huppertz B. (2010). Vasculogenesis and angiogenesis in the endometrium during menstrual cycle and implantation. Acta Histochem..

[39] Grzechocińska B., Dąbrowski F., Cyganek A., Panek G., Wielgoś M. (2017). The role of metalloproteinases in endometrial remodelling during menstrual cycle. Ginekol. Pol..

[40] Sun X., Cheng G., Hao M., Zheng J., Zhou X., Zhang J., Taichman R.S., Pienta K.J., Wang J. (2010). CXCL12/CXCR4/CXCR7 chemokine axis and cancer progression. Cancer Metastasis Rev..

[41] Liu P., Long P., Huang Y., Sun F., Wang Z. (2016). CXCL12/CXCR4 axis induces proliferation and invasion in human endometrial cancer. Am. J. Transl. Res..

[42] Gelmini S., Mangoni M., Castiglione F., Beltrami C., Pieralli A., Andersson K.L., Fambrini M., Taddei G.L., Serio M., Orlando C. (2009). The CXCR4/CXCL12 axis in endometrial cancer. Clin. Exp. Metastasis.

[43] Sahoo S., Zhang X., Hondermarck H., Tanwar P. (2018). The Emerging Role of the Microenvironment in Endometrial Cancer. Cancers.

[44] Guo F., Wang Y., Liu J., Mok S.C., Xue F., Zhang W. (2015). CXCL12/CXCR4: A symbiotic bridge linking cancer cells and their stromal neighbors in oncogenic communication networks. Oncogene.

[45] Felix A.S., Weissfeld J., Edwards R., Linkov F. (2010). Future directions in the field of endometrial cancer research: The need to investigate the tumor microenvironment. Eur. J. Gynaecol. Oncol..

[46] Jackson H.W., Defamie V., Waterhouse P., Khokha R. (2017). TIMPs: Versatile extracellular regulators in cancer. Nat. Rev. Cancer.

[47] Graesslin O., Cortez A., Fauvet R., Lorenzato M., Birembaut P., Daraï E. (2006). Metalloproteinase-2, -7 and -9 and tissue inhibitor of metalloproteinase-1 and -2 expression in normal, hyperplastic and neoplastic endometrium: A clinical-pathological correlation study. Ann. Oncol..

[48] Zhang C., Yang W., Zhang S., Zhang Y., Liu P., Li X., Zhi W., Yang D., Li M., Lu Y. (2022). Pan-cancer analysis of osteogenesis imperfecta causing gene SERPINF1. Intractable Rare Dis. Res..

[49] Brook N., Brook E., Dass C.R., Chan A., Dharmarajan A. (2020). Pigment Epithelium-Derived Factor and Sex Hormone-Responsive Cancers. Cancers.

[50] Risques R.A., Kennedy S.R. (2018). Aging and the rise of somatic cancer-associated mutations in normal tissues. PLoS Genet..

[51] Milholland B., Auton A., Suh Y., Vijg J. (2015). Age-related somatic mutations in the cancer genome. Oncotarget.

[52] Catasus L., Pons C., Muñoz J., Espinosa I., Prat J. (2013). Promoter hypermethylation contributes to TIMP3 down-regulation in high stage endometrioid endometrial carcinomas. Histopathology.

[53] Shaw E., Farris M., McNeil J., Friedenreich C., Pischon T., Nimptsch K. (2016). Obesity and Endometrial Cancer. Obesity and Cancer. Recent Results in Cancer Research.

[54] Reeves G.K., Pirie K., Beral V., Green J., Spencer E., Bull D. (2007). Cancer incidence and mortality in relation to body mass index in the Million Women Study: Cohort study. Br. Med. J..

[55] Onstad M.A., Schmandt R.E., Lu K.H. (2016). Addressing the role of obesity in endometrial cancer risk, prevention, and treatment. J. Clin. Oncol..

[56] Renehan A.G., Tyson M., Egger M., Heller R.F., Zwahlen M. (2008). Body-mass index and incidence of cancer: A systematic review and meta-analysis of prospective observational studies. Lancet.

[57] Daley-Brown D., Oprea-Ilies G.M., Lee R., Pattillo R., Gonzalez-Perez R.R. (2015). Molecular cues on obesity signals, tumor markers and endometrial cancer. Horm. Mol. Biol. Clin Investig..

[58] Mantzoros C.S., Magkos F., Brinkoetter M., Sienkiewicz E., Dardeno T.A., Kim S.Y., Hamnvik O.P., Koniaris A. (2011). Leptin in human physiology and pathophysiology. Am. J. Physiol. Metab..

[59] Bouloumié A., Drexler H.C., Lafontan M., Busse R. (1998). Leptin, the product of Ob gene, promotes angiogenesis. Circ. Res..

[60] Sierra-Honigmann M.R., Nath A.K., Murakami C., García-Cardeña G., Papapetropoulos A., Sessa W.C., Madge L.A., Schechner J.S., Schwabb M.B., Polverini P.J. (1998). Biological action of leptin as an angiogenic factor. Science.

[61] Ellis P.E., Barron G.A., Bermano G. (2020). Adipocytokines and their relationship to endometrial cancer risk: A systematic review and meta-analysis. Gynecol. Oncol..

[62] Wang P.P., He X.Y., Wang R., Wang Z., Wang Y.G. (2014). High leptin level is an independent risk factor of endometrial cancer: A meta-analysis. Cell Physiol. Biochem..

[63] Hazelwood E., Sanderson E., Tan V.Y., Ruth K.S., Frayling T.M., Dimou N., Gunter M.J., Dossus L., Newton C., Ryan N. (2022). Identifying molecular mediators of the relationship between body mass index and endometrial cancer risk: A Mendelian randomization analysis. BMC Med..

[64] Boroń D., Nowakowski R., Grabarek B.O., Zmarzły N., Opławski M. (2021). Expression pattern of leptin and its receptors in endometrioid endometrial cancer. J. Clin. Med..

[65] Kim C.S., Park H.S., Kawada T., Kim J.H., Lim D., Hubbard N.E., Kwon B.S., Erickson K.L., Yu R. (2006). Circulating levels of MCP-1 and IL-8 are elevated in human obese subjects and associated with obesity-related parameters. Int. J. Obes..

[66] Ciortea R., Mihu D., Mihu C.M. (2014). Association between visceral fat, IL-8 and endometrial cancer. Anticancer Res..

[67] Koch A.E., Polverini P.J., Kunkel S.L., Harlow L.A., DiPietro L.A., Elner V.M., Elner S.G., Strieter R.M. (1992). Interleukin-8 as a Macrophage-Derived Mediator of Angiogenesis. Science.

[68] Fujimoto J., Aoki I., Khatun S., Toyoki H., Tamaya T. (2002). Clinical implications of expression of interleukin-8 related to myometrial invasion with angiogenesis in uterine endometrial cancers. Ann. Oncol..

[69] Kotowicz B., Fuksiewicz M., Jonska-Gmyrek J., Berezowska A., Radziszewski J., Bidzinski M., Kowalska M. (2017). Clinical significance of pretreatment serum levels of VEGF and its receptors, IL- 8, and their prognostic value in type I and II endometrial cancer patients. PLoS ONE.

[70] Raudenska M., Sztalmachova M., Gumulec J., Fojtu M., Polanska H., Balvan J., Feith M., Binkova H., Horakova Z., Kostrica R. (2015). Prognostic significance of the tumour-adjacent tissue in head and neck cancers. Tumor. Biol..

[71] Kulinczak M., Sromek M., Panek G., Zakrzewska K., Lotocka R., Szafron L.M., Chechlinska M., Siwicki J.K. (2022). Endometrial Cancer-Adjacent Tissues Express Higher Levels of Cancer-Promoting Genes than the Matched Tumors. Genes.

[72] Aran D., Camarda R., Odegaard J., Paik H., Oskotsky B., Krings G., Goga A., Sirota M., Butte A.J. (2017). Comprehensive analysis of normal adjacent to tumor transcriptomes. Nat. Commun..

[73] Huang X., Stern D.F., Zhao H. (2016). Transcriptional Profiles from Paired Normal Samples Offer Complementary Information on Cancer Patient Survival—Evidence from TCGA Pan-Cancer Data. Sci. Rep..

[74] Frost H.R. (2021). Analyzing cancer gene expression data through the lens of normal tissue-specificity. PLoS Comput. Biol..

