# Supplementary material for: Decreased Gene Expression of Antiangiogenic Factors in Endometrial Cancer: qPCR Analysis and Machine Learning Modelling
Source: Cancers (Basel). 2023 Jul 18;15(14):3661. doi: 10.3390/cancers15143661 (PMC10378066; doi:10.3390/cancers15143661)
Supplement: Supplementary file 1 [file cancers-15-03661-s001.zip › cancers-2479194-supplementary.pdf]

Table S1: Normalized expression of angiogenesis-associated genes in endometrial cancer tissues and adjacent control tissues. Endometrial cancer tissue, n= up to 548; adjacent control tissue, n= up to 35; paired samples, n= up to 23. Data are from the GDC TCGA Endometrioid Cancer (UCEC) study, downloaded from UCSC Xena server [1]. FC, fold change; FR, fold-regulation.

GENE	tissue	AVERAGE (log2 value)	EXPRESSION (antilog value)	FC (tumor/normal)	**FR (tumor/normal)**	p-value†		Adjusted p-value‡
ADAMTS1	**normal**	19.884	967565.632	0.2	**-4.2**	<0.0001	****	0.0092
	**tumor**	17.808	229433.889					
AMOT	**normal**	16.761	111094.554	0.8	**-1.2**	0.6811	ns	1.0000
	**tumor**	16.494	92322.701					
ANG	**normal**	17.497	184923.805	0.4	**-2.5**	0.0024	*	0.1983
	**tumor**	16.193	74896.438					
ANGPT1	**normal**	13.808	14339.571	0.2	**-5.0**	<0.0001	****	0.0092
	**tumor**	11.491	2878.010					
ANGPT2	**normal**	15.133	35928.900	1.4	**1.4**	0.1818	ns	1.0000
	**tumor**	15.590	49315.816					
ANGPT4	**normal**	12.618	6285.075	0.0	**-20.0**	0.0001	***	0.0092
	**tumor**	8.293	313.577					
ANGPTL1	**normal**	17.757	221530.046	0.0	**-42.0**	<0.0001	****	0.0092
	**tumor**	12.366	5279.936					
ANGPTL2	**normal**	18.737	437046.897	0.4	**-2.5**	0.0053	*	0.3867
	**tumor**	17.416	174899.651					
ANGPTL3	**normal**	8.191	292.284	0.6	**-1.7**	0.8462	ns	1.0000
	**tumor**	7.439	173.486					
ANGPTL4	**normal**	16.188	74672.309	2.2	**2.2**	0.0010	**	0.0879
	**tumor**	17.294	160743.159					
CD44	**normal**	19.461	721465.773	0.5	**-2.0**	0.0018	*	0.1527
	**tumor**	18.482	366248.545					
CDH5	**normal**	17.758	221661.702	0.5	**-2.1**	0.0059	*	0.4198
	**tumor**	16.692	105871.339					
CEACAM1	**normal**	15.650	51408.321	2.1	**2.1**	0.0013	*	0.1128
	**tumor**	16.753	110452.435					
CHGA	**normal**	13.658	12929.945	4.9	**4.9**	0.0903	ns	0.9998
	**tumor**	15.954	63468.443					
COL15A1	**normal**	18.769	446583.811	0.3	**-3.6**	0.0003	**	0.0272
	**tumor**	16.926	124553.420					
COL18A1	**normal**	19.830	932019.103	1.4	**1.4**	0.1445	ns	1.0000
	**tumor**	20.346	1332877.847					
COL4A1	**normal**	19.460	721037.259	1.3	**1.3**	0.1375	ns	1.0000
	**tumor**	19.829	931052.923					
COL4A2	**normal**	20.637	1630470.765	0.9	**-1.1**	0.5824	ns	1.0000
	**tumor**	20.482	1464920.062					
COL4A3	**normal**	11.909	3846.304	0.3	**-3.5**	0.0021	*	0.1759
	**tumor**	10.088	1088.272					
CSF3	**normal**	13.165	9183.501	0.4	**-2.2**	0.5399	ns	1.0000
	**tumor**	12.010	4123.395					
CXCL10	**normal**	15.957	63605.202	3.6	**3.6**	0.0301	*	0.9399
	**tumor**	17.801	228368.422					
CXCL12	**normal**	19.761	888756.433	0.1	**-16.8**	<0.0001	****	0.0092
	**tumor**	15.690	52854.045					
CXCL2	**normal**	16.282	79668.086	0.6	**-1.7**	0.0360	*	0.9657
	**tumor**	15.543	47742.343					
CXCL8	**normal**	14.040	16840.281	4.4	**4.4**	0.0163	*	0.7795
	**tumor**	16.172	73816.282					
EDIL3	**normal**	16.950	126607.207	0.2	**-4.8**	0.0006	***	0.0537
	**tumor**	14.675	26155.267					
ENPP2	**normal**	19.099	561584.214	0.2	**-5.9**	<0.0001	****	0.0092
	**tumor**	16.534	94865.434					
EPHB2	**normal**	14.342	20766.925	1.8	**1.8**	0.0475	*	0.9886
	**tumor**	15.188	37333.766					
F2	**normal**	7.274	154.803	3.8	**3.8**	0.0462	*	0.9871
	**tumor**	9.184	581.475					
FBLN5	**normal**	19.774	896712.333	0.2	**-4.7**	<0.0001	****	0.0092
	**tumor**	17.533	189668.482					
FGA	**normal**	5.820	56.506	1.2	**1.2**	0.4038	ns	1.0000
	**tumor**	6.110	69.082					
FGF1	**normal**	14.245	19410.793	0.6	**-1.6**	0.1404	ns	1.0000
	**tumor**	13.560	12078.374					
FGF2	**normal**	17.137	144114.617	0.1	**-17.5**	<0.0001	****	0.0092

	**tumor**	13.011	8256.851					
FGF4	**normal**	1.657	3.153	17.6	**17.6**	0.0002	**	0.0182
	**tumor**	5.797	55.601					
FLT1	**normal**	16.605	99668.994	0.6	**-1.5**	0.2861	ns	1.0000
	**tumor**	15.981	64680.363					
FLT4	**normal**	16.689	105665.351	0.2	**-4.4**	<0.0001	****	0.0092
	**tumor**	14.542	23857.166					
FN1	**normal**	19.485	734004.087	0.5	**-1.9**	0.0005	**	0.0450
	**tumor**	18.585	393101.896					
FOXC2	**normal**	12.487	5740.770	0.1	**-7.4**	0.0004	***	0.0361
	**tumor**	9.591	771.094					
FST	**normal**	14.996	32677.274	0.3	**-3.1**	0.0027	*	0.2202
	**tumor**	13.341	10378.578					
GAPDH	**normal**	23.141	9248943.477	3.3	**3.3**	<0.0001	****	0.0092
	**tumor**	24.852	30278073.390					
GRN	**normal**	20.833	1868103.370	2.2	**2.2**	<0.0001	****	0.0092
	**tumor**	21.985	4150920.873					
GUSB	**normal**	18.738	437133.460	1.3	**1.3**	0.0026	*	0.2130
	**tumor**	19.099	561562.702					
HEY1	**normal**	15.225	38302.285	1.9	**1.9**	0.0135	*	0.7136
	**tumor**	16.124	71419.620					
HGF	**normal**	14.429	22055.596	0.1	**-7.2**	0.0049	**	0.3636
	**tumor**	11.571	3042.247					
HPRT1	**normal**	18.324	328215.925	1.6	**1.6**	<0.0001	****	0.0092
	**tumor**	18.972	514135.221					
HSPG2	**normal**	18.820	462790.589	0.6	**-1.8**	<0.0001	****	0.0092
	**tumor**	17.982	258976.830					
IFNB1	**normal**	2.488	5.611	3.0	**3.0**	0.8040	ns	1.0000
	**tumor**	4.072	16.824					
IFNG	**normal**	8.361	328.765	3.7	**3.7**	0.8117	ns	1.0000
	**tumor**	10.258	1224.777					
IL12A	**normal**	13.049	8472.497	0.5	**-2.1**	0.0059	**	0.4198
	**tumor**	11.978	4032.885					
ITGA4	**normal**	13.497	11562.317	1.0	**1.0**	0.6705	ns	1.0000
	**tumor**	13.553	12019.608					
ITGAV	**normal**	18.555	385171.880	0.5	**-2.0**	<0.0001	****	0.0092
	**tumor**	17.559	193137.673					
ITGB3	**normal**	14.869	29926.643	0.3	**-3.8**	<0.0001	****	0.0092
	**tumor**	12.935	7830.747					
KDR	**normal**	17.923	248544.191	0.2	**-4.5**	<0.0001	****	0.0092
	**tumor**	15.749	55062.305					
KIT	**normal**	15.574	48770.268	0.3	**-3.1**	0.0027	**	0.2202
	**tumor**	13.920	15500.503					
LEP	**normal**	7.345	162.618	0.5	**-1.8**	0.4977	ns	1.0000
	**tumor**	6.459	87.976					
LYVE1	**normal**	15.873	60031.353	0.1	**-8.1**	<0.0001	****	0.0092
	**tumor**	12.852	7395.362					
MDK	**normal**	21.547	3063728.752	2.8	**2.8**	<0.0001	****	0.0092
	**tumor**	23.034	8587542.294					
MMP2	**normal**	20.978	2065005.632	0.5	**-2.1**	0.1601	ns	1.0000
	**tumor**	19.933	1000658.484					
NRP1	**normal**	18.221	305630.410	0.4	**-2.7**	<0.0001	****	0.0092
	**tumor**	16.798	113950.478					
NRP2	**normal**	17.681	210203.705	0.4	**-2.7**	<0.0001	****	0.0092
	**tumor**	16.244	77609.283					
PDGFB	**normal**	17.273	158392.320	0.8	**-1.3**	0.0709	ns	0.9988
	**tumor**	16.946	126233.030					
PDGFRA	**normal**	17.943	251963.834	0.1	**-7.8**	<0.0001	****	0.0092
	**tumor**	14.981	32335.355					
PDGFRB	**normal**	19.571	778934.697	0.3	**-3.5**	<0.0001	****	0.0092
	**tumor**	17.759	221870.958					
PECAM1	**normal**	18.770	447026.242	0.5	**-2.2**	0.0060	**	0.4252
	**tumor**	17.655	206348.908					
PF4	**normal**	8.441	347.573	1.3	**1.3**	0.9240	ns	1.0000
	**tumor**	8.780	439.655					
PLG	**normal**	9.464	706.443	0.4	**-2.4**	0.0006	***	0.0537
	**tumor**	8.179	289.783					
PRL	**normal**	9.380	666.353	0.8	**-1.2**	0.9854	ns	1.0000
	**tumor**	9.123	557.511					
PROK1	**normal**	10.540	1488.603	0.4	**-2.5**	0.7928	ns	1.0000
	**tumor**	9.198	587.189					

PROX1	**normal**	14.241	19368.553	0.2	**-4.2**	0.0007	***	0.0624
	**tumor**	12.187	4663.570					
PTN	**normal**	19.439	710688.162	0.2	**-4.8**	0.0112	*	0.6452
	**tumor**	17.176	148033.656					
S1PR1	**normal**	18.052	271818.800	0.2	**-4.1**	<0.0001	****	0.0092
	**tumor**	16.031	66968.087					
SEMA3F	**normal**	17.583	196282.419	2.0	**2.0**	0.0109	*	0.6352
	**tumor**	18.550	383743.030					
SERPINB5	**normal**	10.203	1178.412	7.1	**7.1**	0.0384	*	0.9727
	**tumor**	13.021	8310.591					
SERPINC1	**normal**	9.437	693.139	1.2	**1.2**	0.0228	*	0.8802
	**tumor**	9.700	831.528					
SERPINF1	**normal**	21.185	2384786.316	0.2	**-6.1**	<0.0001	****	0.0092
	**tumor**	18.574	390110.120					
TEK	**normal**	17.063	136936.058	0.2	**-5.1**	0.0003	***	0.0272
	**tumor**	14.715	26886.578					
TGFA	**normal**	14.359	21006.838	3.8	**3.8**	0.0003	***	0.0272
	**tumor**	16.301	80712.861					
TGFB1	**normal**	18.861	476084.173	0.7	**-1.5**	0.0035	**	0.2757
	**tumor**	18.276	317385.328					
THBS1	**normal**	20.091	1117178.723	0.2	**-5.2**	<0.0001	****	0.0092
	**tumor**	17.707	213909.694					
THBS2	**normal**	18.506	372272.623	0.2	**-4.5**	<0.0001	****	0.0092
	**tumor**	16.343	83126.981					
TIE1	**normal**	17.787	226140.073	0.3	**-3.4**	0.0002	***	0.0182
	**tumor**	16.035	67163.194					
TIMP2	**normal**	21.578	3131203.221	0.2	**-5.0**	<0.0001	****	0.0092
	**tumor**	19.246	621943.223					
TIMP3	**normal**	15.705	53421.870	0.1	**-13.6**	<0.0001	****	0.0092
	**tumor**	11.940	3927.894					
TNF	**normal**	12.937	7844.608	4.1	**4.1**	0.2861	ns	1.0000
	**tumor**	14.957	31798.550					
TNFSF15	**normal**	12.790	7080.887	2.5	**2.5**	0.0980	ns	0.9999
	**tumor**	14.106	17628.212					
TNMD	**normal**	10.790	1770.783	0.1	**-12.7**	0.0163	*	0.7795
	**tumor**	7.127	139.797					
TNNI1	**normal**	12.152	4552.183	2.7	**2.7**	0.0060	**	0.4252
	**tumor**	13.595	12370.755					
TYMP	**normal**	16.785	112958.129	3.4	**3.4**	0.0484	*	0.9896
	**tumor**	18.556	385435.889					
VASH1	**normal**	16.613	100243.062	0.5	**-1.9**	<0.0001	****	0.0092
	**tumor**	15.658	51721.819					
VEGFA	**normal**	17.265	157547.635	1.5	**1.5**	0.0049	**	0.3636
	**tumor**	17.896	243967.951					
VEGFB	**normal**	20.491	1473542.400	0.9	**-1.1**	0.7202	ns	1.0000
	**tumor**	20.405	1388565.916					
VEGFC	**normal**	17.635	203486.200	0.4	**-2.6**	0.0001	***	0.0092
	**tumor**	16.248	77807.831					

†Wilcoxon matched-pairs signed rank test; ‡ Bonferroni-Šidák corrections for multiple comparisons

Table S2: Normalized levels of angiogenesis-associated proteins in endometrial cancer tissues and adjacent control tissues (n= up to 527). Data are from the CPTAC UCEC Discovery Study – Proteome, PDC ID: PDC000125 [2]. FC, fold change; FR, fold-regulation.

PROTEIN	tissue	AVERAGE (log2 value)	PROTEIN LEVEL (antilog value)	FC (tumor/normal)	FR (tumor/normal)	P-VALUE†		Adjusted p value‡
**ADAMTS1**	**normal**	0.099	**1.071**	1.0	**1.0**	**0.1974**	**ns**	1.0000
	**tumor**	0.122	**1.088**					
**AMOT**	**normal**	0.314	**1.243**	0.8	**-1.3**	**0.0150**	*****	0.6141
	**tumor**	-0.068	**0.954**					
**ANGPT2**	**normal**	0.077	**1.055**	1.0	**-1.0**	**0.9530**	**ns**	1.0000
	**tumor**	0.038	**1.027**					
**ANGPT4**	**normal**	/	**/**	/	**/**	**/**		
	**tumor**	-0.457	**0.729**					
**ANGPTL1**	**normal**	0.606	**1.522**	0.6	**-1.8**	**0.1094**	**ns**	0.9993
	**tumor**	-0.247	**0.843**					
**ANGPTL2**	**normal**	1.273	**2.417**	0.3	**-3.0**	**<0.0001**	********	0.0063
	**tumor**	-0.309	**0.807**					
**ANGPTL4**	**normal**	0.045	**1.032**	1.0	**1.0**	**0.0210**	*****	0.7374
	**tumor**	0.073	**1.052**					
**CD44**	**normal**	0.053	**1.037**	0.9	**-1.1**	**0.6231**	**ns**	1.0000
	**tumor**	-0.078	**0.947**					
**CDH5**	**normal**	0.575	**1.489**	0.5	**-1.9**	**<0.0001**	********	0.0063
	**tumor**	-0.366	**0.776**					
**CEACAM1**	**normal**	-0.366	**0.776**	1.4	**1.4**	**<0.0001**	********	0.0063
	**tumor**	0.122	**1.088**					
**CHGA**	**normal**	-0.031	**0.979**	1.6	**1.6**	**0.1074**	**ns**	0.9992
	**tumor**	0.618	**1.534**					
**COL15A1**	**normal**	2.227	**4.681**	0.2	**-6.2**	**<0.0001**	********	0.0063
	**tumor**	-0.404	**0.756**					
**COL18A1**	**normal**	1.528	**2.884**	0.3	**-3.5**	**<0.0001**	********	0.0063
	**tumor**	-0.265	**0.832**					
**COL4A1**	**normal**	0.808	**1.751**	0.4	**-2.3**	**<0.0001**	********	0.0063
	**tumor**	-0.364	**0.777**					
**COL4A2**	**normal**	0.484	**1.399**	0.6	**-1.8**	**<0.0001**	********	0.0063
	**tumor**	-0.324	**0.799**					
**CTGF**	**normal**	0.447	**1.363**	0.7	**-1.5**	**0.0031**	******	0.1777
	**tumor**	-0.100	**0.933**					
**CXCL10**	**normal**	-0.106	**0.929**	1.3	**1.3**	**0.0391**	*****	0.9190
	**tumor**	0.226	**1.170**					
**CXCL12**	**normal**	2.612	**6.112**	0.1	**-8.2**	**<0.0001**	********	0.0063
	**tumor**	-0.427	**0.744**					
**CXCL8**	**normal**	-0.274	**0.827**	1.6	**1.6**	**0.0457**	*****	0.9475
	**tumor**	0.406	**1.325**					
**EDIL3**	**normal**	0.590	**1.505**	0.6	**-1.6**	**0.0034**	******	0.1931
	**tumor**	-0.088	**0.941**					
**ENPP2**	**normal**	0.826	**1.773**	0.4	**-2.3**	**<0.0001**	********	0.0063
	**tumor**	-0.397	**0.760**					
**EPHB2**	**normal**	-0.229	**0.853**	1.2	**1.2**	**0.0395**	*****	0.9211
	**tumor**	-0.022	**0.985**					
**FBLN5**	**normal**	1.463	**2.757**	0.2	**-4.0**	**<0.0001**	********	0.0063
	**tumor**	-0.553	**0.682**					
**FGA**	**normal**	0.377	**1.298**	0.6	**-1.6**	**0.0035**	******	0.1982
	**tumor**	-0.289	**0.819**					
**FGF1**	**normal**	0.505	**1.419**	0.6	**-1.6**	**<0.0001**	********	0.0063
	**tumor**	-0.187	**0.879**					
**FGF2**	**normal**	2.263	**4.800**	0.1	**-7.4**	**<0.0001**	********	0.0063
	**tumor**	-0.617	**0.652**					
**FGF4**	**normal**	-0.292	**0.817**	1.8	**1.8**	**0.2500**	**ns**	1.0000
	**tumor**	0.564	**1.478**					
**FLT1**	**normal**	0.533	**1.447**	0.6	**-1.6**	**<0.0001**	********	0.0063
	**tumor**	-0.113	**0.925**					
**FN1**	**normal**	0.464	**1.379**	0.6	**-1.6**	**0.0211**	*****	0.7391
	**tumor**	-0.222	**0.857**					

**FST**	**normal**	1.556	**2.940**	0.2	**-4.8**	**<0.0001**	********	0.0063
	**tumor**	-0.707	**0.613**					
**GRN**	**normal**	-0.307	**0.808**	1.4	**1.4**	**<0.0001**	********	0.0063
	**tumor**	0.167	**1.122**					
**HGF**	**normal**	1.377	**2.597**	0.3	**-3.7**	**<0.0001**	********	0.0063
	**tumor**	-0.494	**0.710**					
**HSPG2**	**normal**	1.611	**3.055**	0.3	**-3.7**	**<0.0001**	********	0.0063
	**tumor**	-0.265	**0.832**					
**ITGA4**	**normal**	-0.023	**0.984**	1.1	**1.1**	**0.0965**	**ns**	0.9983
	**tumor**	0.070	**1.050**					
**ITGAV**	**normal**	-0.277	**0.825**	1.1	**1.1**	**0.0087**	******	0.4233
	**tumor**	-0.079	**0.946**					
**ITGB3**	**normal**	0.156	**1.115**	0.8	**-1.2**	**0.3165**	**ns**	1.0000
	**tumor**	-0.155	**0.898**					
**LEP**	**normal**	0.485	**1.399**	0.6	**-1.6**	**0.0938**	**ns**	0.9980
	**tumor**	-0.191	**0.876**					
**LYVE1**	**normal**	1.286	**2.439**	0.3	**-3.2**	**<0.0001**	********	0.0063
	**tumor**	-0.371	**0.773**					
**MDK**	**normal**	-0.081	**0.946**	1.2	**1.2**	**0.0048**	******	0.2615
	**tumor**	0.190	**1.141**					
**MMP2**	**normal**	0.741	**1.672**	0.5	**-2.2**	**0.0001**	*******	0.0063
	**tumor**	-0.382	**0.767**					
**NRP1**	**normal**	0.245	**1.185**	0.7	**-1.5**	**<0.0001**	********	0.0063
	**tumor**	-0.373	**0.772**					
**NRP2**	**normal**	0.260	**1.198**	0.8	**-1.3**	**0.0604**	**ns**	0.9803
	**tumor**	-0.090	**0.940**					
**PDGFRA**	**normal**	0.826	**1.773**	0.4	**-2.6**	**0.0313**	*****	0.8651
	**tumor**	-0.557	**0.680**					
**PDGFRB**	**normal**	0.831	**1.779**	0.4	**-2.3**	**<0.0001**	********	0.0063
	**tumor**	-0.361	**0.779**					
**PECAM1**	**normal**	0.551	**1.465**	0.5	**-1.8**	**0.0001**	*******	0.0063
	**tumor**	-0.312	**0.806**					
**PF4**	**normal**	0.311	**1.241**	0.8	**-1.3**	**0.3449**	**ns**	1.0000
	**tumor**	-0.093	**0.938**					
**PLG**	**normal**	1.096	**2.137**	0.3	**-3.1**	**<0.0001**	********	0.0063
	**tumor**	-0.517	**0.699**					
**PROX1**	**normal**	-0.265	**0.832**	1.3	**1.3**	**0.4037**	**ns**	1.0000
	**tumor**	0.062	**1.044**					
**PTN**	**normal**	2.035	**4.097**	0.2	**-6.4**	**<0.0001**	********	0.0063
	**tumor**	-0.646	**0.639**					
**RNASE4**	**normal**	1.610	**3.053**	0.3	**-3.5**	**<0.0001**	********	0.0063
	**tumor**	-0.199	**0.871**					
**SEMA3F**	**normal**	-0.193	**0.875**	1.2	**1.2**	**0.0313**	*****	0.8651
	**tumor**	0.121	**1.087**					
**SERPINB5**	**normal**	-0.183	**0.881**	1.7	**1.7**	**0.0691**	**ns**	0.9890
	**tumor**	0.571	**1.486**					
**SERPINC1**	**normal**	1.913	**3.766**	0.2	**-5.3**	**<0.0001**	********	0.0063
	**tumor**	-0.487	**0.713**					
**SERPINF1**	**normal**	1.399	**2.637**	0.3	**-3.3**	**<0.0001**	********	0.0063
	**tumor**	-0.329	**0.796**					
**TGFA**	**normal**	0.394	**1.314**	0.6	**-1.5**	**0.9999**	**ns**	1.0000
	**tumor**	-0.236	**0.849**					
**TGFB1**	**normal**	0.162	**1.119**	0.8	**-1.3**	**0.0025**	******	0.1459
	**tumor**	-0.184	**0.880**					
**THBS1**	**normal**	0.428	**1.345**	0.7	**-1.4**	**0.2799**	**ns**	1.0000
	**tumor**	-0.014	**0.991**					
**THBS2**	**normal**	0.489	**1.404**	0.7	**-1.4**	**0.0025**	******	0.1459
	**tumor**	-0.043	**0.970**					
**TIMP2**	**normal**	1.088	**2.126**	0.4	**-2.7**	**<0.0001**	********	0.0063
	**tumor**	-0.367	**0.775**					
**TIMP3**	**normal**	2.170	**4.499**	0.2	**-5.6**	**<0.0001**	********	0.0063
	**tumor**	-0.325	**0.798**					
**TYMP**	**normal**	-0.807	**0.572**	2.3	**2.3**	**<0.0001**	********	0.0063
	**tumor**	0.412	**1.331**					
**VASH1**	**normal**	-0.011	**0.993**	1.0	**1.0**	**0.9515**	**ns**	1.0000
	**tumor**	0.058	**1.041**					

**VEGFA**	**normal**	-0.271	**0.829**	1.2	**1.2**	**0.1424**	**ns**	0.9999
	**tumor**	0.004	**1.003**					
**VEGFB**	**normal**	0.513	**1.427**	0.7	**-1.4**	**0.0313**	*****	0.8651
	**tumor**	-0.012	**0.992**					

†Wilcoxon matched-pairs signed rank test; ‡ Bonferroni-Šidák corrections for multiple comparisons

Table S3: Expression of angiogenesis-associated genes in endometrial cancer tissue (n= up to 527). Data are from the TCGA Pan-Cancer study [3].

Gene	**mRNA expression**, RSEM (Batch normalized from Illumina HiSeq_RNASeqV2)	SD	Gene	**mRNA expression**, RSEM (Batch normalized from Illumina HiSeq_RNASeqV2)	SD
MDK	18267.75	14206.90	*TIE1*	395.18	351.54
COL18A1	16148.50	11747.65	*KDR*	390.16	342.72
COL4A2	12913.68	8832.25	*ANGPT2*	386.21	350.38
GRN	12674.28	7719.52	*S1PR1*	250.72	221.36
FN1	10472.11	19006.95	*ITGA4*	237.83	296.47
HSPG2	9320.44	6691.21	*ANG*	235.19	258.93
COL4A1	8674.58	7144.93	*FLT4*	198.86	165.24
MMP2	7478.64	8846.14	*ITGB3*	189.88	546.20
CD44	4761.79	7723.18	*VEGFC*	181.54	154.98
TIMP2	4469.03	3400.49	*CXCL2*	179.02	351.07
VEGFA	3642.50	2980.86	*KIT*	157.84	284.90
CHGA	3347.87	13011.92	*TEK*	143.39	159.57
CCN2	3099.48	3530.17	*SERPINB5*	136.73	307.67
ITGAV	2241.80	1577.89	*FGF2*	127.44	175.78
THBS1	2221.83	2725.16	*TNF*	127.01	254.38
ADAMTS1	1933.68	2453.26	*CSF3*	120.73	606.01
PDGFRB	1865.27	1670.35	*TNNI1*	94.68	144.80
SEMA3F	1814.86	1550.70	*ANGPTL1*	78.98	188.42
TIMP3	1811.47	2514.89	*FGF1*	77.94	89.96
NRP1	1686.45	1357.42	*EDIL3*	57.34	82.69
VEGFB	1618.82	949.99	*ANGPT1*	46.48	181.63
SERPINF1	1611.30	1804.28	*FST*	42.82	132.45
TYMP	1293.09	1450.48	*ADGRB1*	42.79	119.20
NRP2	1089.81	1191.26	*COL4A3*	36.83	153.65
FBLN5	1080.58	1195.57	*PROX1*	36.57	132.80
COL15A1	1077.84	1153.32	*LYVE1*	29.87	26.20
AMOT	1054.77	1195.04	*FGA*	22.54	132.17
CEACAM1	967.82	1031.49	*TNFSF15*	20.68	25.90
FLT1	918.18	723.83	*IL12A*	19.09	43.44
ENPP2	820.21	1093.42	*HGF*	17.60	45.26
TGFB1	810.16	646.44	*TNMD*	15.53	234.67
THBS2	754.77	1132.91	*PLG*	13.60	84.03
PECAM1	730.22	562.79	*F2*	11.62	87.36
ANGPTL2	718.82	582.33	*IFNG*	10.78	34.88
ANGPTL4	715.81	1180.25	*CNMD*	9.33	59.13
PDGFRA	674.74	1096.33	*PROK1*	6.59	21.46
TGFA	655.51	797.61	*VEGFD*	6.31	13.08
EPHB2	645.82	729.06	*PRL*	5.63	35.03
CDH5	602.33	481.09	*FOXC2*	5.45	13.20
CXCL10	545.38	903.37	*PF4*	3.93	11.92
CXCL12	540.60	743.52	*ANGPT4*	3.35	11.83
PTN	529.22	1027.89	*SERPINC1*	2.57	9.40
PDGFB	503.11	372.73	*FGF4*	2.25	15.45
VASH1	479.33	298.00	*IFNB1*	1.52	5.15
CXCL8	424.51	693.92	*ANGPTL3*	1.10	2.94
RNASE4	421.10	457.71	*LEP*	0.91	2.55
HEY1	408.30	640.72			

Figure S1: Volcano plots identifying significant changes in gene expression within the tumour and tumour-adjacent tissue separately, in EC patients stratified according to the presence of LVI or DMI, EC grade, FIGO stage, and menopausal status.

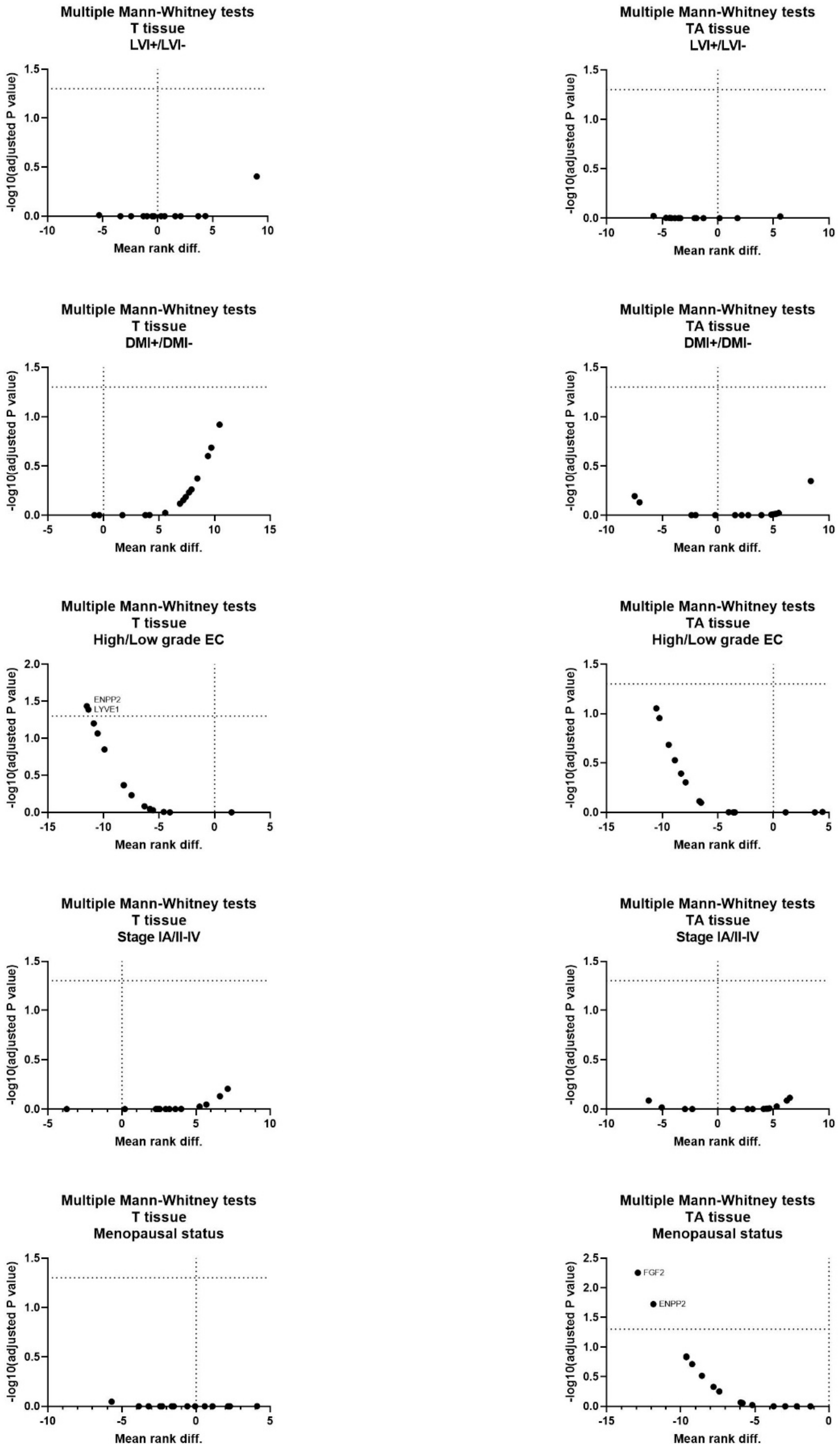

Table S4: The calculated distribution fit correlation with the original data, including the p-values and estimated upper and lower bounds for the 95% confidence intervals for the TCGA dataset. All results are significant at $p < 0.001$.

DATA POINT	A	B	CORRELATION COEFFICIENT	P-VALUE	LOWER BOUND	UPPER BOUND
**T_IL8**	0.865526	0.002878	0.905640	0.0000	0.821542	0.951169
**T_CXCL12**	0.794219	0.000954	0.789788	0.0000	0.622871	0.887929
**T_FGF2**	1.071074	0.000068	0.675944	0.0000	0.446581	0.821948
**T_LEP**	2.084855	0.000000	0.819681	0.0000	0.672152	0.904610
**T_LYVE1**	0.630536	0.000004	0.605505	0.0001	0.345750	0.779062
**T_NRP1**	1.581737	0.000020	0.830570	0.0000	0.690435	0.910623
**T_TIMP2**	1.476134	0.000019	0.847401	0.0000	0.719050	0.919850
**T_TIMP3**	0.666441	0.000051	0.778116	0.0000	0.603981	0.881345
**T_CSF3**	0.718792	0.000000	0.858897	0.0000	0.738849	0.926106
**T_ENPP2**	0.884903	0.000169	0.879080	0.0000	0.774116	0.937001
**T_FBLN5**	1.199094	0.000026	0.917302	0.0000	0.842739	0.957330
**T_FST**	0.642057	0.000000	0.768969	0.0000	0.589314	0.876158
**T_PDGFRB**	1.519883	0.000012	0.815921	0.0000	0.665881	0.902526
**T_TEK**	1.075751	0.000006	0.774892	0.0000	0.598798	0.879520
**T_SERPINF1**	1.082361	0.000075	0.912356	0.0000	0.833721	0.954722
**TA_IL8**	0.714176	0.001788	0.821890	0.0000	0.675846	0.905833
**TA_CXCL12**	2.376653	0.001271	0.955058	0.0000	0.912992	0.977030
**TA_FGF2**	2.363325	0.000102	0.927692	0.0000	0.861822	0.962789
**TA_LEP**	2.999550	0.000000	0.731613	0.0000	0.530617	0.854710
**TA_LYVE1**	1.505071	0.000004	0.920799	0.0000	0.849141	0.959171
**TA_NRP1**	4.049333	0.000015	0.871276	0.0000	0.760402	0.932802
**TA_TIMP2**	3.801149	0.000026	0.923189	0.0000	0.853529	0.960427
**TA_TIMP3**	2.644840	0.000046	0.987744	0.0000	0.975896	0.993787

**TA_CSF3**	0.535299	0.000001	0.646848	0.0000	0.404212	0.804432
**TA_ENPP2**	2.019394	0.000241	0.957371	0.0000	0.917379	0.978225
**TA_FBLN5**	3.357543	0.000027	0.974838	0.0000	0.950822	0.987203
**TA_FST**	1.933152	0.000000	0.933093	0.0000	0.871816	0.965615
**TA_PDGFRB**	7.527304	0.000006	0.945569	0.0000	0.895098	0.972114
**TA_TEK**	3.541413	0.000004	0.956357	0.0000	0.915455	0.977702
**TA_SERPINF1**	5.229769	0.000047	0.945371	0.0000	0.894726	0.972011

Table S5: The calculated distribution fit correlation with the original data, including the p-values and estimated upper and lower bounds for the 95% confidence intervals for the study dataset. All results are significant at $p < 0.001$.

DATA POINT	A	B	CORRELATION COEFFICIENT	P-VALUE	LOWER BOUND	UPPER BOUND
**T_IL8**	0.689577	615.611904	0.733600	0.0000	0.691516	0.770720
**T_CXCL12**	0.982751	550.091291	0.746537	0.0000	0.706152	0.782082
**T_FGF2**	1.001729	127.220793	0.749339	0.0000	0.709326	0.784539
**T_LEP**	0.139387	9.920558	0.486866	0.0000	0.418872	0.549431
**T_LYVE1**	1.703864	17.531297	0.829551	0.0000	0.800884	0.854424
**T_NRP1**	1.862639	905.409032	0.874982	0.0000	0.853343	0.893611
**T_TIMP2**	2.115066	2112.948146	0.892638	0.0000	0.873850	0.908764
**T_TIMP3**	1.201862	1507.220707	0.728567	0.0000	0.685833	0.766295
**T_CSF3**	0.180336	669.781675	0.310728	0.0000	0.231458	0.385899
**T_ENPP2**	1.076607	761.844598	0.753251	0.0000	0.713760	0.787968
**T_FBLN5**	1.323874	816.222788	0.792192	0.0000	0.758073	0.821987
**T_FST**	0.314102	137.296118	0.493572	0.0000	0.426123	0.555563
**T_PDGFRB**	1.851507	1007.434155	0.823138	0.0000	0.793515	0.848870
**T_TEK**	1.454318	98.592971	0.749232	0.0000	0.709204	0.784445
**T_SERPINF1**	1.210736	1330.840563	0.809321	0.0000	0.777665	0.836883
**TA_IL8**	0.489189	352.721598	0.701191	0.0003	0.396924	0.866606
**TA_CXCL12**	0.831011	9572.143563	0.941913	0.0000	0.863030	0.975953
**TA_FGF2**	6.445643	202.961500	0.985955	0.0000	0.965830	0.994262
**TA_LEP**	0.279601	11.188484	0.794556	0.0000	0.560817	0.910991
**TA_LYVE1**	1.050753	534.399004	0.825833	0.0000	0.620146	0.925279
**TA_NRP1**	3.446996	1012.546962	0.964297	0.0000	0.914473	0.985319
**TA_TIMP2**	1.590801	13860.605010	0.964402	0.0000	0.914719	0.985362
**TA_TIMP3**	1.238316	15977.840950	0.973597	0.0000	0.936330	0.989173
**TA_CSF3**	0.202173	1278.145710	0.730288	0.0001	0.446001	0.880725
**TA_ENPP2**	1.024887	4131.928738	0.943870	0.0000	0.867463	0.976776
**TA_FBLN5**	1.608749	2777.478876	0.977029	0.0000	0.944471	0.990590
**TA_FST**	0.999620	62.177321	0.899825	0.0000	0.770538	0.957995
**TA_PDGFRB**	2.085009	2551.811220	0.973161	0.0000	0.935300	0.988993
**TA_TEK**	0.843587	999.543226	0.959695	0.0000	0.903764	0.983403
**TA_SERPINF1**	1.832375	4023.756396	0.950340	0.0000	0.882207	0.979493

Table S6: Comparison between the training and test datasets using the Wilcoxon Rank Sum Test. None of the variables is significantly different between the datasets.

VARIABLE	WILCOXON RANK SUM P-VALUE	
**AGE**	0.7711	ns
**WEIGHT**	0.7443	ns
**GRADESUMMARY**	0.1671	ns
**R_IL8**	0.5794	ns
**R_CXCL12**	0.9348	ns
**R_FGF2**	0.0735	ns
**R_LEP**	0.0920	ns
**R_LYVE1**	0.4293	ns
**R_NRP1**	0.5307	ns
**R_TIMP2**	0.4729	ns
**R_TIMP3**	0.7095	ns
**R_CSF3**	0.8345	ns
**R_ENPP2**	0.3881	ns
**R_FBLN5**	0.4729	ns
**R_FST**	0.4618	ns
**R_PDGFRB**	0.4729	ns
**R_TEK**	0.5072	ns
**R_SERPINF1**	0.3881	ns
**T_IL8_NORM**	0.9348	ns
**T_CXCL12_NORM**	0.7368	ns
**T_FGF2_NORM**	0.5671	ns
**T_LEP_NORM**	0.0855	ns
**T_LYVE1_NORM**	0.5671	ns
**T_NRP1_NORM**	0.7643	ns
**T_TIMP2_NORM**	0.4084	ns
**T_TIMP3_NORM**	0.2716	ns
**T_CSF3_NORM**	0.4508	ns
**T_ENPP2_NORM**	0.3684	ns
**T_FBLN5_NORM**	0.2878	ns
**T_FST_NORM**	0.1160	ns
**T_PDGFRB_NORM**	0.3132	ns
**T_TEK_NORM**	0.1817	ns
**T_SERPINF1_NORM**	0.1877	ns
**TA_IL8_NORM**	0.5427	ns
**TA_CXCL12_NORM**	0.6562	ns
**TA_FGF2_NORM**	0.3132	ns
**TA_LEP_NORM**	0.0574	ns
**TA_LYVE1_NORM**	0.6173	ns
**TA_NRP1_NORM**	0.0827	ns
**TA_TIMP2_NORM**	0.1758	ns
**TA_TIMP3_NORM**	0.3588	ns
**TA_CSF3_NORM**	0.5919	ns
**TA_ENPP2_NORM**	0.6694	ns
**TA_FBLN5_NORM**	0.9204	ns
**TA_FST_NORM**	0.8062	ns

**TA_PDGFRB_NORM**	0.6562	ns
**TA_TEK_NORM**	0.8203	ns
**TA_SERPINF1_NORM**	0.3684	ns
**R_IL8_NORM**	0.9204	ns
**R_CXCL12_NORM**	0.6046	ns
**R_FGF2_NORM**	0.8203	ns
**R_LEP_NORM**	0.9204	ns
**R_LYVE1_NORM**	0.9493	ns
**R_NRP1_NORM**	0.1001	ns
**R_TIMP2_NORM**	0.5189	ns
**R_TIMP3_NORM**	0.6694	ns
**R_CSF3_NORM**	0.9638	ns
**R_ENPP2_NORM**	0.2796	ns
**R_FBLN5_NORM**	0.5548	ns
**R_FST_NORM**	0.1486	ns
**R_PDGFRB_NORM**	0.7922	ns
**R_TEK_NORM**	0.1877	ns

References

1. Goldman, M.J.; Craft, B.; Hastie, M.; Repečka, K.; McDade, F.; Kamath, A.; Banerjee, A.; Luo, Y.; Rogers, D.; Brooks, A.N.; et al. *Visualizing and interpreting cancer genomics data via the Xena platform.* Nature Biotechnology, 2020. **38**(6): p. 675-678. DOI: 10.1038/s41587-020-0546-8.
2. Dou, Y.; Kawaler, E.A.; Cui Zhou, D.; Gritsenko, M.A.; Huang, C.; Blumenberg, L.; Karpova, A.; Petyuk, V.A.; Savage, S.R.; Satpathy, S.; et al. *Proteogenomic Characterization of Endometrial Carcinoma.* Cell, 2020. **180**(4): p. 729-748 e726. DOI: 10.1016/j.cell.2020.01.026.
3. Cancer Genome Atlas Research, N.; Weinstein, J.N.; Collisson, E.A.; Mills, G.B.; Shaw, K.R.; Ozenberger, B.A.; Ellrott, K.; Shmulevich, I.; Sander, C.; and Stuart, J.M. *The Cancer Genome Atlas Pan-Cancer analysis project.* Nat Genet, 2013. **45**(10): p. 1113-1120. DOI: 10.1038/ng.2764.
